# Decision-based models of the implementation of interventions in systems of healthcare: Implementation outcomes and intervention effectiveness in complex service environments

**DOI:** 10.1371/journal.pone.0223129

**Published:** 2019-10-17

**Authors:** Arno Parolini, Wei Wu Tan, Aron Shlonsky

**Affiliations:** 1 Department of Social Work, University of Melbourne, Carlton, Victoria, Australia; 2 Department of Social Work, Monash University, Caulfield East, Victoria, Australia; Flinders University, AUSTRALIA

## Abstract

Implementation is a crucial component for the success of interventions in health service systems, as failure to implement well can have detrimental impacts on the effectiveness of evidence-based practices. Therefore, evaluations conducted in real-world contexts should consider how interventions are implemented and sustained. However, the complexity of healthcare environments poses considerable challenges to the evaluation of interventions and the impact of implementation efforts on the effectiveness of evidence-based practices. In consequence, implementation and intervention effectiveness are often assessed separately in health services research, which prevents the direct investigation of the relationships of implementation components and effectiveness of the intervention. This article describes multilevel decision juncture models based on advances in implementation research and causal inference to study implementation in health service systems. The multilevel decision juncture model is a theory-driven systems approach that integrates structural causal models with frameworks for implementation. This integration enables investigation of interventions and their implementation within a single model that considers the causal links between levels of the system. Using a hypothetical youth mental health intervention inspired by published studies from the health service research and implementation literature, we demonstrate that such theory-based systems models enable investigations of the causal pathways between the implementation outcomes as well as their links to patient outcomes. Results from Monte Carlo simulations also highlight the benefits of structural causal models for covariate selection as consistent estimation requires only the inclusion of a minimal set of covariates. Such models are applicable to real-world context using different study designs, including longitudinal analyses which facilitates the investigation of sustainment of interventions.

## Introduction

Identifying the barriers, facilitators and causal mechanisms that affect successful implementation of complex interventions is crucial for understanding their potential effects in different contexts [1–4]. After a decade of implementation research, we are now at a stage where barriers and facilitators to adopting evidence-based practices (EBPs) have been identified [5], scales to measure these factors have been developed [6], frameworks that describe these factors in context have been proposed to understand the process of implementation and facilitate implementation planning [7–9]. Additionally, implementation outcomes, distinct from that of intervention effectiveness, have been suggested [10].

Yet, there remain substantial challenges in this area of research, mostly due to the complexity of dynamic systems of care [11]. This has led to the recommendation of focused research efforts in three areas: 1. Scaling up of EBPs to broaden their reach and impact; 2. Addressing multiple levels of changes in service systems; 3. Adoption and sustainment of multiple EBPs by large systems of care, as patients routinely face multiple problems [12].

In past analyses, implementation effectiveness and intervention effectiveness have been investigated together in hybrid designs [13] but they did not attempt to model implementation and intervention as a parts of a single system. Hence, these approaches did not formally account for the causal mechanisms linking different systems components. This presents a considerable gap since in implementation research, knowledge accumulation requires methods for causal inference that consider the complex structure of the systems in which interventions are implemented and investigate the pathways through which implementation works [14,15].

A causal approach to health systems implementation research in real-world contexts brings with it two challenges. First, formal methodologies describing the causal linkages between implementation outcomes and intervention effectiveness are not readily available. Second, rigorous methods for establishing causality in this field have largely been limited to randomized controlled trials (RCT), which makes the identification of causal relationships between implementation outcomes and intervention effectiveness difficult. Moreover, conclusions drawn from RCTs, especially regarding external validity, depend on a series of prerequisites [16] that may be untenable in complex social contexts [3,17–20]. Hence, findings from RCTs may be of limited value for understanding and addressing the barriers to successful implementation of EBPs in real-world contexts in the absence of a cumulative scientific progress [17] based on formal models of how implementation strategies are related to intervention effectiveness.

In this paper, we address issues regarding implementation and intervention effectiveness raised in the recent literature on implementation and health service research. We present implementation as an intervention itself within a complex system where multiple levels are linked through stakeholder decisions at different points. Taking this approach to modeling implementation has several major advantages: (i) it allows us to model implementation components and intervention outcomes as integrated elements of a complex process within a single system, (ii) it enables us to integrate implementation frameworks with structural causal systems [21] to investigate the effects of implementation strategies and outcomes as well as intervention effectiveness, and (iii) it makes all model assumptions explicit, leading to transparency of research.

From a methodological perspective, our model can be interpreted as an extension of hybrid designs [13] by formally considering causal links between implementation and intervention components of the system. To demonstrate how the approach can be applied in practice, we use a hypothetical example based on empirical studies in the health service research literature. We also discuss the implications of this approach for implementation research and the evaluation of complex interventions in systems.

### A decision-based causal model of implementation and intervention effectiveness

The key idea in our approach is that the process of implementation can be structured as a series of conditional decision points or choices. These choices are represented by a set of causal relationships between the decision outcome and characteristics of the inner, outer, and patient contexts, as well as the intervention itself. This results in the implementation, systems, and effectiveness outcomes being treated as integrated and linked parts of a system. By defining the implementation process as a series of decision points, our model puts stakeholders at the center of the system. In other words, barriers, facilitators and other variables related to the implementation and intervention processes take effects through the choices made by stakeholders at different levels of the implementation system, including policy makers, organizations, practitioners, and patients.

### Decision-based implementation systems

**Fig 1:**
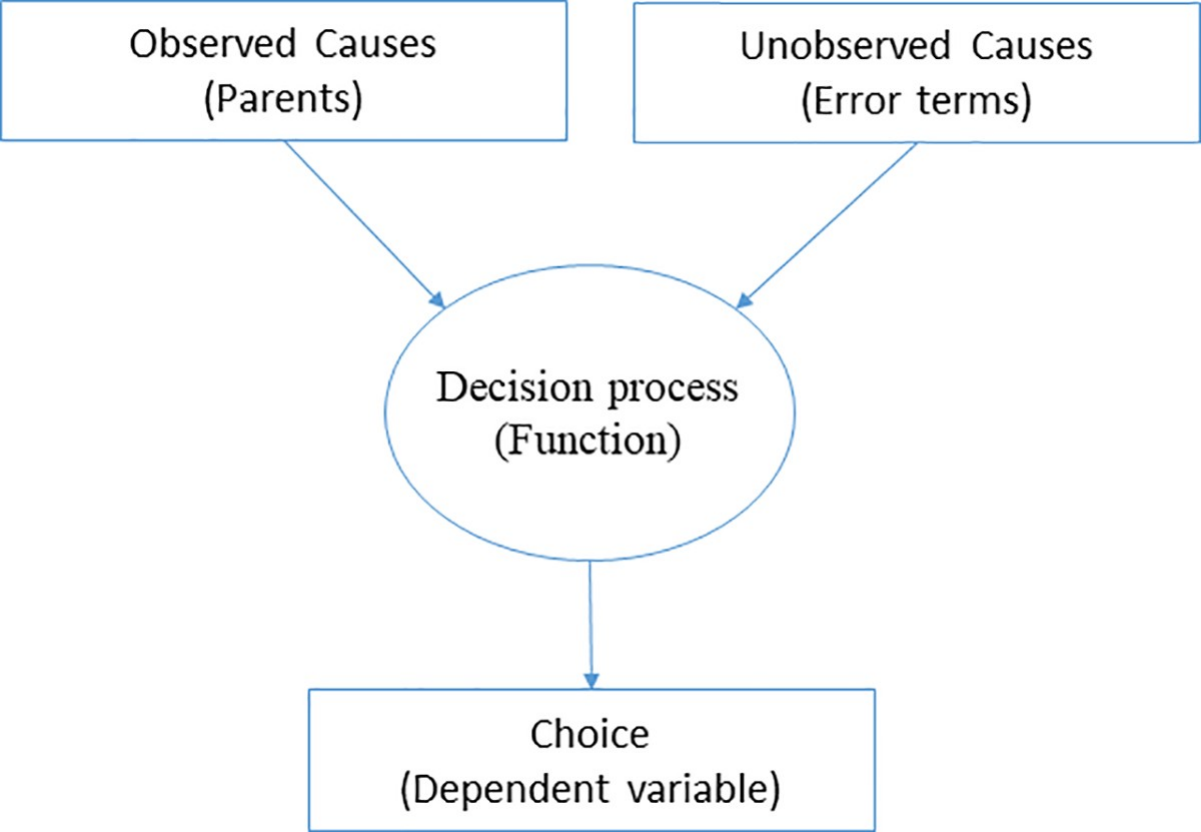
Graphical representation of a structural equation for a single decision point.

To explain the underlying idea of framing the implementation process as a series of decisions, we begin by describing how individual choice situations are formulated within a structural causal model. A structural causal model consists of a set of structural equations. Each equation represents a causal relationship between an outcome, or dependent variable, and the explanatory variables of interest, which are often denoted as ‘parents’ of a particular outcome variable [21]. Applying this informal definition of structural functions to a particular decision node, we can represent a causal equation of each choice with the diagram shown in Fig 1. In the diagram, both observed causes and unobserved causes are factors in the decision process or function which leads to an observed choice, with unobserved causes represented as error terms. Structural models are subject to such error terms or uncertainties since researchers usually cannot observe all the causal factors driving a particular decision. Nevertheless, we can still make probabilistic statements about observed choice processes [22] and these statements have causal interpretation under certain assumptions, as described in the S1 Appendix.

The particular forms of these causal relationships are based on theoretical models that are derived from our understanding of the world. In our case, these theoretical models explain how decisions are made in the implementation process. A thorough treatment of structural equations, especially in a choice context, is beyond the scope of this article and we defer a more detailed exposition of this topic to the S1 Appendix.

As mentioned above, we assume that each person acting within the implementation system is confronted with making at least one choice among different options. For example, a manager in an organization may choose between several implementation strategies and a practitioner can choose alternative interventions that may be appropriate for a particular patient.

An observed choice made by an actor in the system is framed as a result of a comparative process. A decision maker weighs the benefits and costs of each alternative, which are not necessarily measured in monetary units, and compares the net benefits of available options to come to a conclusion. It is exactly the series of such decision processes that determine the pathway of implementation in the system and, consequently, the effectiveness of the intervention.

To integrate the structural causal approach into a decision framework, we must understand the behavioral process leading to decision makers’ observed choices [22]. In other words, we will need to understand what the drivers and barriers are at each decision point in the system. As such, the decision-based implementation approach is based on a theoretical behavioral model of how people make choices which is, in turn, context-specific and should be informed by evidence in implementation research and behavioral theory. The structural causal approach examines how people along the implementation process select one of the alternative pathways at each decision juncture. The emphasis is on learning about what drives people to make these decisions, how different decisions are related to each other, and how these are related to specific, measurable consequences.

In the next step, we will integrate structural decision models with implementation systems using the Exploration, Preparation, Implementation, Sustainment (EPIS) framework [7]. This model has been widely used [23] and it interprets implementation as a process consisting of four phases: (i) Exploration, (ii) Adoption Decision/Preparation, (iii) Active Implementation, and (iv) Sustainment.

The Exploration phase refers to the awareness among organizations of issues to be addressed or service aspects to be improved. This phase transitions to the next when a decision is made by the organizational leadership to implement one or more interventions.

During the Adoption Decision/Preparation phase, organizations search for existing evidence-based innovations or interventions that can be used to address these issues. In this phase the organization sets an implementation goal, weighted against relevant outer context and inner context barriers and facilitators. Implementation strategies which may be a complex plan of implementation involving more than one strategic element or discrete implementation strategy [24] are guided by the organization’s goals as well as balancing the cost of implementation and the anticipated benefits of the intervention(s) [25].

Active Implementation refers to organization-wide application of the interventions. This phase also encompasses practitioners’ decisions to implement and patients actually receiving the interventions. It is therefore the phase where implementation outcomes [10] are situated and linked to intervention effectiveness and service outcomes. For example, feasibility, fidelity, adoption, or acceptability are all directly influenced by decisions made during earlier phases. Moreover, these outcomes are themselves mediators and moderators on the pathways from the choice of implementation strategies over practitioner uptake decisions to patient outcomes.

The potentially iterative nature of implementation has long been recognized [26]. An important concept of the Sustainment phase is the impact of experiences or lessons learned in one implementation project on future iterations of the same project or different implementation efforts [7]. We view implementation as an ongoing iterative process with experiences providing feedback to the system as a form of continuous quality improvement (CQI) of care. Phases of implementation can be fluid in the sense that an organization may revisit preceding phases with new information generated from subsequent phases. CQI becomes a critical part of the implementation process and implementation becomes part of routine practice of an organization through CQI.

**Fig 2:**
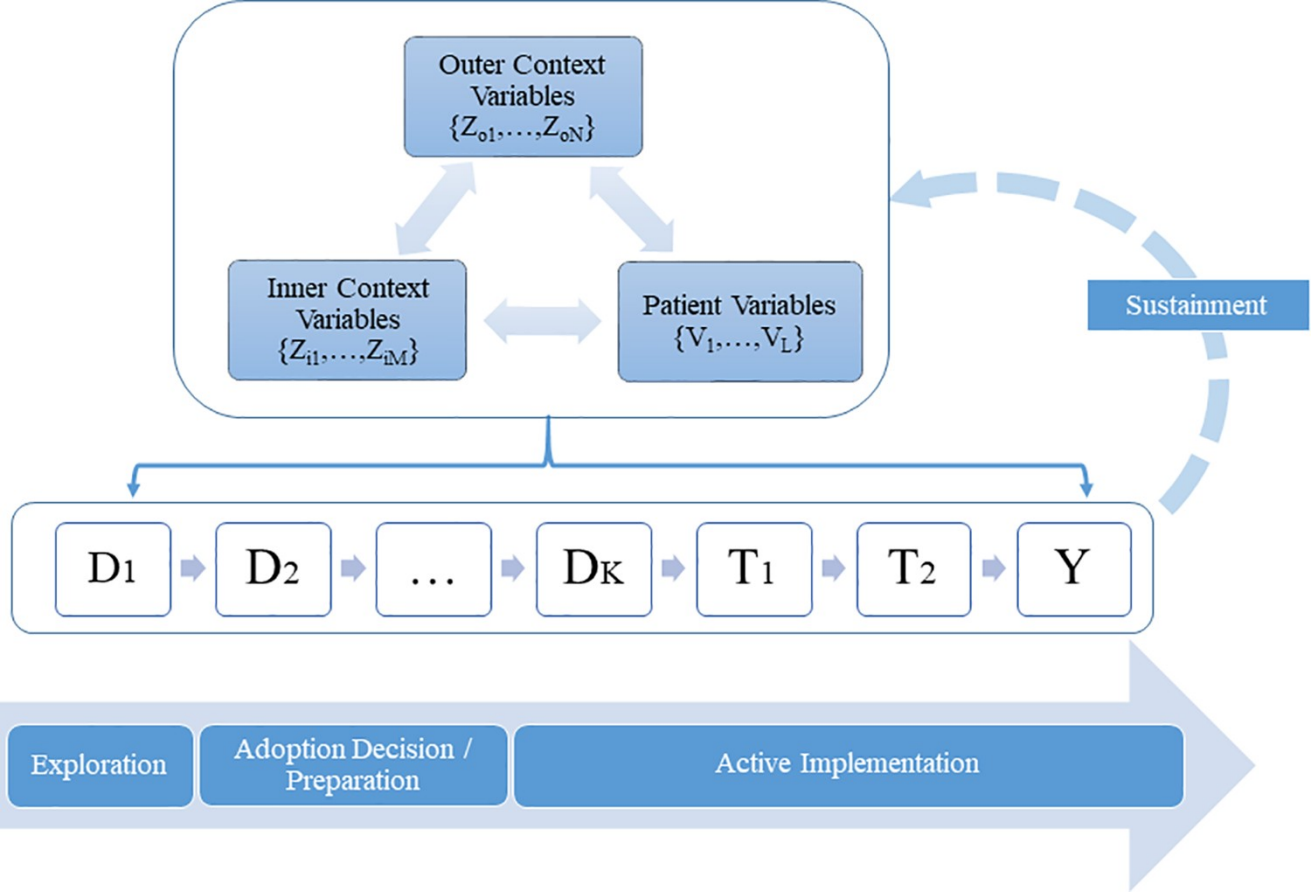
Multilevel decision juncture model.

Fig 2 illustrates the systems approach as a multiple-level decision juncture model. The three boxes at the top of the diagram represent variables of the outer context, inner context, and patients where bi-directional arrows indicate mutual influences among the three sets.

The broad arrow at the bottom of the diagram denotes the first three stages of implementation, with the Sustainment phase depicted as a feedback loop linking outcomes at various levels of the system into the three sets of variables. Decision-making junctures along the organizational decision chain (denoted by the letter D) are located in the middle of the diagram, spanning the phases of implementation. At the right-hand side of the decision-making chain are T_1_, T_2_, and Y, which denote practitioners’ decision to buy-in, patient uptake, and intervention outcome respectively. At each decision juncture, decision makers can choose from a set of alternatives which results in an observed choice outcome as shown in Fig 1. Note that each decision is actually an outcome itself and is conditioned on previous paths.

### Analyzing implementation and intervention effectiveness

Much of implementation research is guided by the paradigm of the Potential Outcomes Framework [27], which is based on idealized experiments [13]. Recent developments in the literature on causal inference have provided unified frameworks [21,28–30] which view counterfactuals as derived from structural systems and define treatment effects within structural causal models. This provides an intuitive representation of the decision processes faced by stakeholders in implementation-intervention systems, allowing us to causally link decision outcomes, implementation outcomes, service outcomes, and patient outcomes in a cohesive model. The phased interpretation of the multilevel decision juncture model for implementation described in the previous section results in a dynamic specification where the choice options and outcomes of one phase depend on the decisions made in previous phases. Researchers may require a fully specified structural model or focus on treatment effects, depending on the research question and their confidence in making functional and structural assumptions about the system [31,32]. Given our focus on intervention and implementation effectiveness, we will concentrate on treatment effects and will not discuss the identification of structural parameters.

We follow a three-stage or three-task process as a general procedure for causal analysis in implementation systems. These stages are (1) defining the counterfactual conditions; (2) identifying causal and non-causal parameters ignoring sampling uncertainties; and (3) statistically estimating parameters using real data [33]. Task 1 sets up the core of the structural model by developing a theoretical map of the implementation system, including causal relations between its elements. This includes defining a set of hypotheticals or counterfactuals, which are queries of the kind: “Given the observed characteristics of a particular individual, organization or setting, what would the outcome have been had variable X been set to value x while all else would have been the same?” [34]. In other words, we are interested in changes of a particular outcome (e.g., patient outcome) had the decision maker (e.g., practitioner) made a different choice (e.g., treatment or implementation strategy) at an earlier point in time. Counterfactuals are derived from structural systems, guided by a precisely formulated scientific theory [21,33]. This theory should reflect our knowledge based on existing research on domains and constructs of implementation such as the Consolidated Framework For Implementation Research (CFIR) [8], the relationships between those elements [14,15], theories and scientific evidence on human behavior [35,36], models for behavior change [37] and qualitative knowledge provided by practitioners, patients and other stakeholders. The theoretical map in this stage determines the structure of the multilevel juncture decision model described above.

Task 2 is concerned with identifying parameters under the assumption of infinitely large samples [21] and considering self-selection of decision makers into pathways. The aim here is to see whether the desired effects can be identified in the given system.

Task 3 introduces statistical uncertainty and is concerned with the estimation of effects using real data. As statistical inference is the goal in this stage, researchers have to consider that they may be working with finite samples rather than population data. Hence controlling for sampling variation, measurement error, design effects (e.g., cluster sampling), or family-wise error rates should be considered in this stage where appropriate. It is also important to note that while the decision models and counterfactuals are defined at the individual levels, the individual treatment effects are generally not identified due to missing information on alternative outcomes for each individual [38]. Nevertheless, as we demonstrate in the hypothetical example and in supporting information, several relevant parameters may be identified, including the average treatment effect or heterogeneous treatment effects across different subpopulations (S1 Appendix).

### A hypothetical example with simulation

We now demonstrate the structural decision approach using a simplified case example of cognitive behavioral therapy (CBT) for anxiety disorders in children and adolescents. Although hypothetical, the context is inspired by studies published in the area of implementation of mental health interventions in allied healthcare settings [39–41] and it is important to note that this multilevel decision juncture model can be applied to any healthcare setting.

In this scenario, an individual-format of cognitive behavioral therapy is funded by a state government for delivery by mental health service providers, including community agencies and hospitals. Existing evidence shows that CBT is an effective treatment for anxiety disorders in child and adolescent patient populations [39,40] and the government decided to bring the therapy to scale, introducing it at a regional level.

However, the evidence of the comparative advantage of CBT to other treatments and its long-term effectiveness are mixed [42] and therefore perceptions on the state of evidence vary among organizational decision makers in our example. Organizations are free to choose to adopt CBT to treat anxiety or to provide a different treatment (e.g., service as usual). The CBT program can be implemented in one of two ways, either without (basic strategy) or with facilitated support structures, such as workshops, that incur costs on the organizations (enhanced strategy). We further assume that existing evidence suggests that the enhanced strategy will lead to improved implementation outcomes.

**Fig 3:**
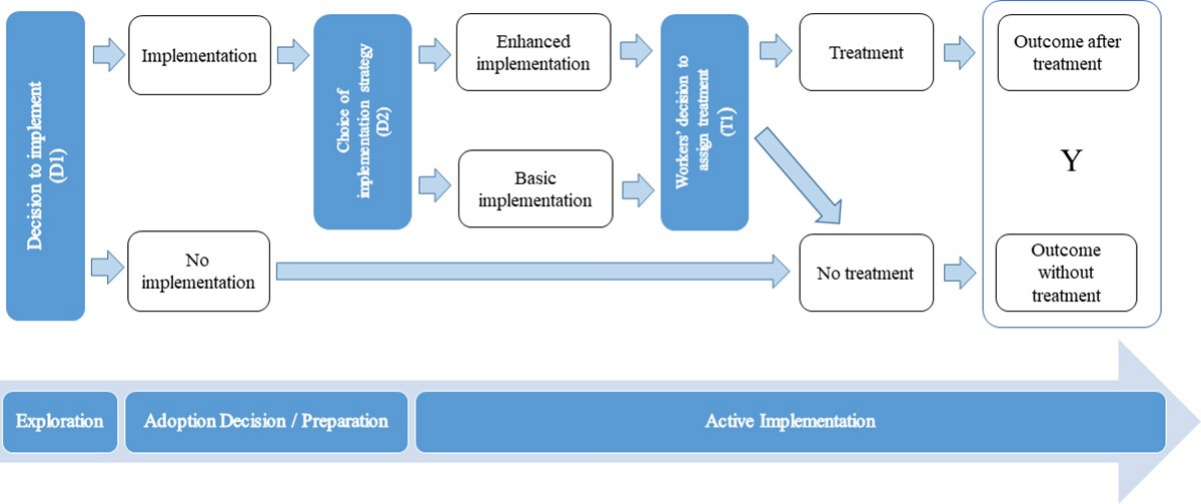
Flow chart for a hypothetical study of the implementation of CBT.

Fig 3 depicts a flow chart of the case example, with decision junctures mapped out through the first three phases of implementation–Exploration, Adoption Decision/Preparation, and Active Implementation. The colored boxes show the three main decision junctures. In the Exploration phase, organizations will choose whether to implement or not (D1 = 1 or 0) based on their expected net benefits from the intervention. In the Adoption Decision/Planning phase, they will choose the implementation strategy that will maximize their net benefits or utilities (D2 = 1 for enhanced or 0 for basic).

Further along, implementation is rolled out and practitioners decide on whether to assign individual patients to the CBT treatment or not (T1 = 1 or 0). In this example, patients are assumed to comply with treatment protocols although this could be included in the model by creating another decision juncture. In a final step, treatment affects patients’ outcomes (Y). The decisions incorporate a complex choice process that is influenced by patient and caseworker characteristics as well as restrictions imposed by the system (e.g. time restrictions, equipment or limited budget).

This hypothetical case study is deliberately presented in a structure that facilitates comparison of traditional approaches with decision-based structural models, which link outcomes at different levels of the system through causal pathways. For example, in a RCT, unit-level randomization would be situated at decision node T1 in Fig 3. In a Hybrid Type 3 study [13], randomization would occur at decision node D2 and a cluster-randomized study focused on treatment effectiveness would be situated at decision node D1. This highlights a significant advantage of decision-based structural systems–they can be seen as theory-driven systems models that extend traditional approaches, such as hybrid designs, to enable learning about how different elements of the system affect outcomes through the chain of decisions, in line with the cumulative scientific progress [17].

The process of analysis can proceed iteratively. As we learn more about the system, we also understand how to effectively sustain and improve the implementation, which may change what we do and the decisions we make as we go. However, our example, as an illustration of the approach, excludes feedback and dynamic effects and we also assume that patients do not influence each other (i.e., patients’ outcomes are mutually independent).

We now apply the three-stage process to investigate effects of interest using techniques described in the literature on causal inference [21,43]. With a broad audience in mind, we refrain from providing technical details or definitions and refer readers to the supporting information and references therein (S1 Appendix).

#### Task 1: Defining hypotheticals or counterfactuals

Guided by clearly specified research questions, the first step is to define the counterfactuals. In this example, we investigate the effect of an intervention and implementation strategy which sets the causal variables of interest (e.g., the implementation strategy) to a specific value, holding all other variables constant.

**Fig 4:**
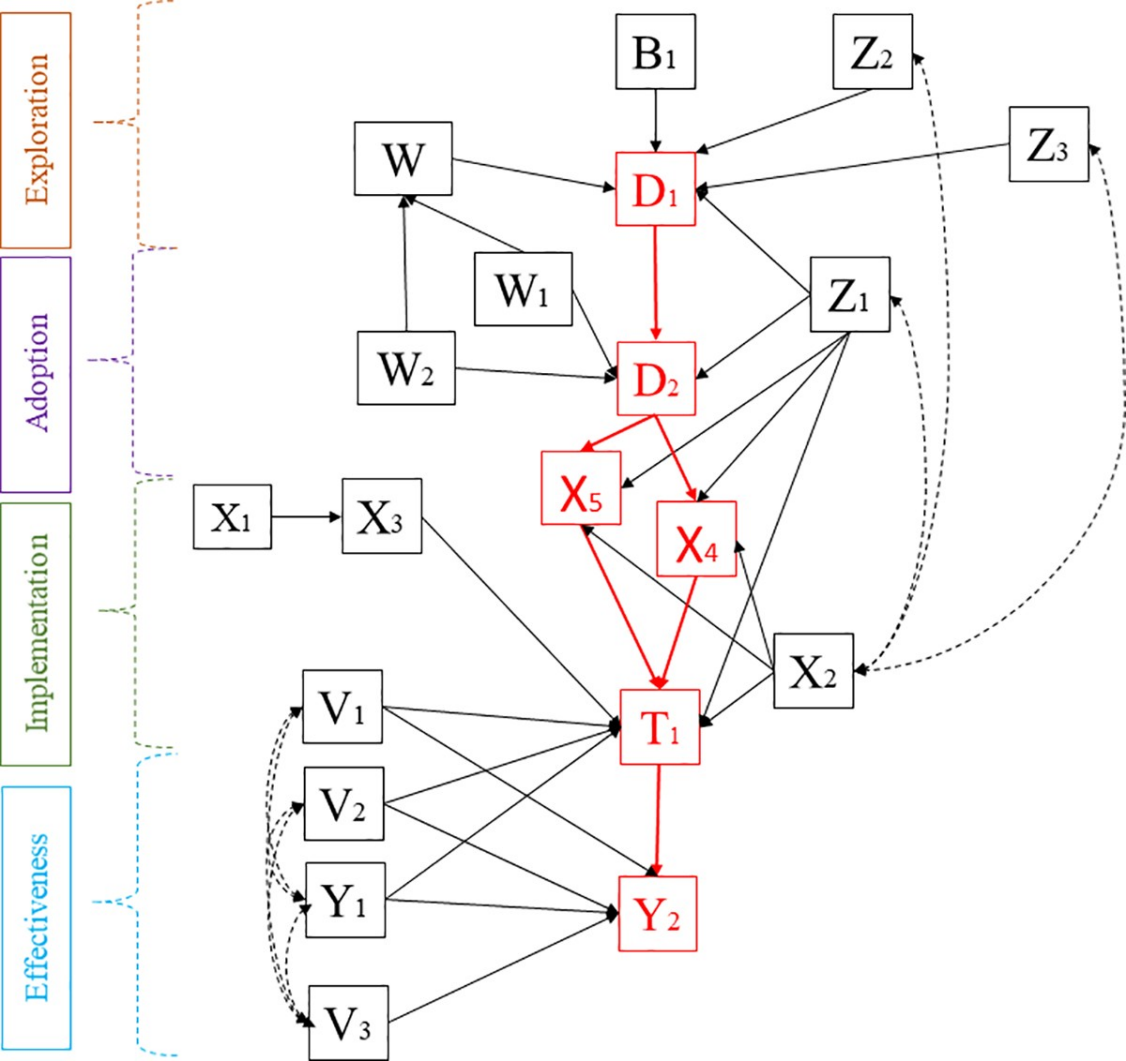
Directed acyclic graph for a hypothetical implementation of CBT.

Based on theory, existing evidence, practical knowledge and available data, researchers generate a structural model of the intervention system. We assume that the following variables are included in the analysis (Fig 4):

- Organizational variables–intra-organizational networks (Z_1_), management style (Z_2_), and organizational structure (Z_3_).
- Implementation- and intervention-specific characteristics–anticipated costs of each implementation strategy (W_1,_ W_2_), an aggregate cost variable (W) and empirical evidence of effectiveness (B_1_).
- Practitioner variables–age (X_1_), perceived leadership in organization (X_2_), tenure in current job (X_3_), perceived feasibility of the treatment (X_4_), and perceived appropriateness of the treatment (X_5_).
- Patient variables–age (V_1_), gender (V_2_), socio-economic status (V_3_), patient outcome scores at baseline (Y_1_) and follow-up (Y_2_).

Given our assumptions for this example, we formulate the system using a directed acyclic graph (DAG) [21]. DAGs present the causal system in nonparametric form [21] and have the advantage of being more accessible for our purpose than systems of equations (see S1 Appendix).

Fig 4 shows the DAG of the structural causal model for this hypothetical example. All assumptions about causal mechanisms are made explicit in the graph (see S1 Appendix). Solid unidirectional arrows represent a causal effect from one variable to another (e.g. organizational variable Z_1_ and cost of strategy W_1_ are parents to decision-node D_2_) and dashed bi-directional arrows represent unobserved correlated errors between two variables. The absence of an arrow between variables implies the absence of direct causal relationships [21]. Each node also has an unobserved error as input, which, per convention, are not shown in the graph and are assumed to be mutually independent [21].

D_1_, D_2_ and T_1_ are decision nodes as in Fig 3. Note that practitioners in organizations not implementing CBT do not participate in subsequent choices. In this example, outcome variables at baseline (Y_1_) and follow-up (Y_2_) are assumed to be continuous assessment scores.

From an implementation perspective, variables representing feasibility (X_4_), appropriateness (X_5_), and practitioners’ decision to adopt the intervention by assigning patients to treatment (T_1_) are of particular interest as they represent examples of implementation outcomes in the system [10]. While these variables are measured at the practitioner level, their distributions (e.g., averages, percentiles, etc.) can be interpreted as implementation outcomes regarding whether a particular intervention was perceived as being feasible (X_4_) or appropriate (X_5_) and whether the intervention was adopted by practitioners (T_1_). The model presented in Fig 4 directly illustrates how implementation strategies (D_2_) are linked with implementation outcomes (X_4_, X_5_, T_1_), which in turn mediate the pathways to patient outcomes (Y_2_) and hence intervention effectiveness. Furthermore, the model also highlights how implementation outcomes influence each other, as emphasized in the literature [10].

#### Task 2: Identification of effects

Based on Fig 4, we can investigate whether parameters of interests are identified in the model using theorems stated in the causal inference literature, the references to which are provided in the S1 Appendix.

For example, the effect of the implementation strategy (D_2_) on perceived feasibility (X_4_) and appropriateness (X_5_) can be identified by controlling for intra-organizational networks (Z_1_) and perceived leadership (X_2_). The effect of feasibility on the probability of treatment assignment (T_1_) is identified by controlling for variables Z_1_, X_2_ and X_5_ as justified by the back-door criterion [21]. While X_3_, V_1_, V_2_ and Y_1_ are not necessary for identification of this effect, inclusion will generally increase precision of estimation [43]. The effect of X_5_ on T_1_ is identified using a similar approach.

Finally, identification of the treatment effect of T_1_ on the outcomes score at follow-up (Y_2_) requires control for case characteristics such as age (V_1_), gender (V_2_), and the baseline score (Y_1_) as they are all confounders. From an efficiency perspective, V_3_ should also be considered as a covariate [43].

Following this approach, we can try to identify any relationship expressed in the graph as is discussed in more detail in the S1 Appendix. These examples highlight how structural models facilitate the selection of variables to be included for estimation by making assumptions about the causal relationships explicit.

#### Task 3: Statistical estimation

The final task is to estimate the effects of interest from actual data. We demonstrate this using Monte-Carlo simulations. These simulations are based on a designed dataset that has predetermined characteristics and a specified structure that optimally approximates a real scenario [44]. Since the true structure of the model is known in simulations, they allow us to show that using the decision juncture model combined with a structural causal approach, we are indeed able to obtain the estimated effects in an implementation system. Demonstrating the appropriateness of such an approach is not possible in experimental or observational studies as we do not have information about the true structure of the system.

In the present study, data are created from a data-generating process that follows the structure shown in Fig 4. The generated dataset is intended to approximate a realistic scenario, where each organization employs several professionals who each work with several patients. This results in a hierarchical dataset that resembles the structure generally observed in allied healthcare settings, including mental health services [41]. The overall sample size for the simulations was set to 6000 patients, which is well within the range of government administrative databases in reality. This sample size was chosen to avoid problems arising from the hierarchical structure of the data or low statistical power. However, structural decision models can also be applied to data with smaller sample sizes as often observed in the implementation literature [41] and it is important to note that Tasks 1 and 2 are independent of sample size.

As the estimates retrieved from a single sample can vary substantially, we run the simulated model 10000 times, which reveals whether the estimated effects are consistent (i.e., unbiased in large samples) and whether statistical inference based on these estimates is reliable.

Each repetition represents an analysis using a different sample. In other words, we estimate each of the effects discussed in Task 2 many times and then analyze the results over all 10000 repetitions. A detailed description of the assumptions for the simulations as well as the Stata code used to conduct the simulation is available in the S1 Appendix.

**Fig 5:**
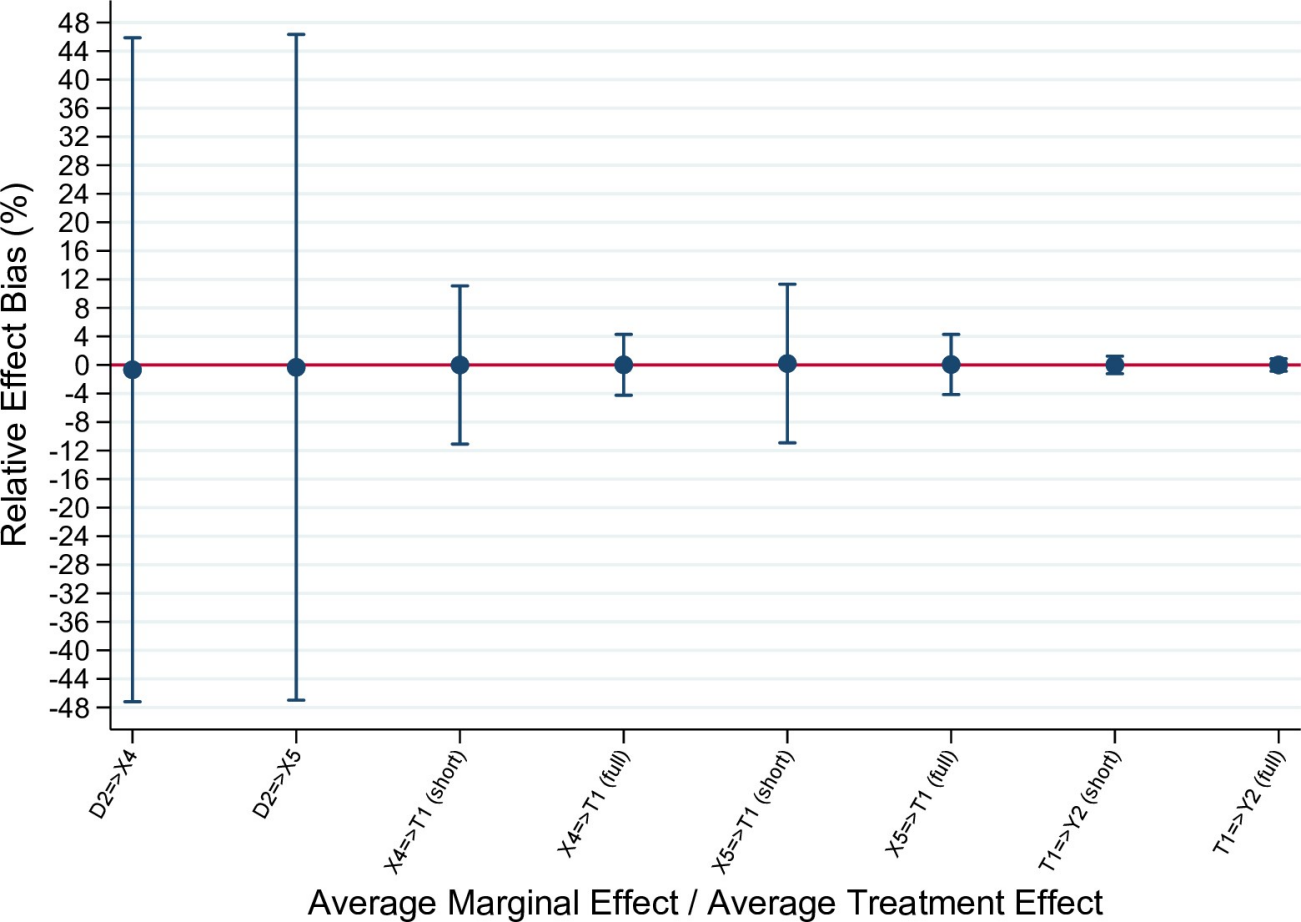
Results from Monte Carlo simulations–Relative effect bias.

For the sake of simplicity, the functional forms of causal relationships were specified so that average effects in the model can be estimated using standard methods such as linear least squares and probit regressions. As the purpose of this simulation is to demonstrate that the structural causal approach can be used to retrieve estimates of the true effects, we focus on the bias of the estimates over the 10000 repetitions. Fig 5 shows the relative bias for estimated average effects as described in Task 2 above. The arrow in the parameter name on the abscissa indicates the direction of the effect. For the effect of feasibility (X_4_) and appropriateness (X_5_) on treatment probability (T_1_), as well as the effect of T_1_ on patient outcome (Y_2_), we conducted estimations using two models–short specifications and full specifications. The short specifications model includes only variables that are necessary to control for confounding [21] while the full specifications model includes all parent variables of the dependent variable, which increases the precision of the estimate. The relative bias estimates shown in the figure are based on the true average effects observed in the generated sample for each of the 10000 repetitions. The 95 per cent confidence intervals are based on the empirical standard error of the statistic [44,45].

The results show that the estimated average effects are very close to the true effects with the average relative bias being less than one percent for each parameter. Furthermore, the 95 per cent confidence intervals include zero for all effects [45]. However, the results also show significant variation in some of the estimates. Particularly, the effects of implementations strategies (D2) on feasibility and acceptability exhibit large variations. A major contributor to this variation in estimates is the smaller sample sizes as these implementation outcomes vary at organization-level only (see S1 Appendix).

Additionally, the short regressions, which include only the covariates necessary to block back-door paths, exhibit significantly higher variability than the fully specified models, which corroborates our theoretical discussion above. This hypothetical example highlights how structural models can serve to provide justification for critical assumptions made in our estimation models, such as unconfoundedness [30].

In the S1 Appendix, we provide further details as well as results that show the consistency of the parameters rather than average treatment or marginal effects. Evidence that the multilevel structure of the data can be appropriately considered in the models is also shown.

## Discussion

The approach described in this paper frames implementation as a series of decisions at different levels of complex systems. Doing so contributes to implementation and intervention research in multiple ways: (i) learning about causal pathways and dynamic processes in implementation systems, (ii) providing a rigorous and flexible methodology to facilitate evaluation of implementation and intervention effectiveness in real world contexts, and (iii) enhance transparency of research by making all model assumptions explicit.

### Learning about implementation systems and enhancing transparency of research

Recent developments in the literature on causal inference and structural systems have increasingly recognized that the structural approach and the experimental ideal are not necessarily at odds but should be rather seen as complementary [17,32,46]. An advantage of interpreting treatment effects within structural systems is that all assumptions about causal relationships are made explicit [47] and are derived from a theoretical model. This understanding is especially critical for quasi-experimental studies, which do not have the qualities of randomized studies in controlling for confounding causes [32].

A theory-based approach enables us to link the process of implementation with the effectiveness of interventions in a cohesive and logically structured systems model. Guided by theory, structural causal models enable us to gain insight into individual decision processes and, consequently, the direct and indirect pathways through which implementation strategies and interventions are chosen and work within the system, thus contributing to the cumulative scientific progress [17]. Generally, these pathways cannot be tracked in RCTs without relying on structural assumptions because they are, by definition, randomized out [33].

By integrating implementation frameworks [7], existing research on implementation facilitators and barriers [8] and the mechanisms of implementation [14] into structural causal models, this study provides a decision-based approach to structural analysis for implementation research. Causal relationships in the theoretical model specify which constructs and variables enter the system at each decision point. By generating a structural causal model, all assumptions about the system are made explicit and critical assumptions, such as conditional exogeneity, can be justified and may even be tested in some cases [34,48]. This enhances the transparency of implementation research.

It is important to note that the structural causal models described here are fundamentally different from statistical approaches used for descriptive modeling and prediction [49–51]. A major difference is that structural models are based on theory while predictive modeling is generally data-driven. Whereas descriptive models are usually concerned with associations or predicting outcomes, structural models as illustrated in this paper focus on the causal interpretation of effect sizes [50–52]. By framing implementation within a decision-based structural system, we aim to answer complex counterfactual questions about hypothetical states in the system [34] that generally cannot be evaluated through statistical conditioning [21,28]. Taking these differences between predictive, descriptive and structural models into consideration, it becomes evident that only theory-driven models facilitate the identification of causal pathways through the system, reveal optimal leverage points for interventions and CQI efforts, and estimate their expected impacts.

### Facilitating implementation research in real world contexts

Framing implementation systems as sequential chains of decisions emphasizes collaboration between researchers, practitioners and patients during model development and improvement. This is because the models and decision processes have to be made explicit and this requires input from everyone involved in service delivery and receipt.

The structural causal approach also offers some practical advantages for analysts engaged in evaluation research as it consolidates the knowledge about systems in structural models, enabling the estimation of intervention and implementation effects from observational data [21]. For example, the CIFR [8] prescribes five domains with 39 constructs that are critical for the success of implementation. Rather than controlling for all potential variables in every equation, structural models provide guidance in variable selection [30,43]. This emphasizes a focus on parsimonious models [34] and prevents the inclusion of inadequate variables in analytical models [53,54]. Hence, structural causal models provide a guide to efficient data collection and estimation, thus reducing the burden on stakeholders and research budgets [43]. Recent developments in the econometric literature also integrate flexible machine learning approaches with causal models for cases where the number of confounding variables is large, functional specifications are questionable, or the interest lies in heterogeneous treatment effects [52].

The ability to employ data collected by administrative database systems and surveys provides new avenues of causal investigations for implementation research. Administrative datasets provide large sample sizes and often have longer observation periods than data collected specifically for research purposes. Employing high-quality observational data in combination with structural models allows researchers to learn about long-term dynamic effects of interventions and their relation to stakeholders’ choices [32]. Because structural models focus on the selection mechanisms in systems, they may, under certain conditions, also be used to predict impacts of existing interventions in new settings or new interventions in existing settings [33], which is a major goal of implementation research [1].

In order for the causal approach to be successful, it is important that relevant data are collected in sufficient quality [55]. Integrating implementation with CQI and research may provide the impetus to rethink data collection to include measures of implementation elements [8,56] in order to support structural analysis [55].

Our approach emphasizes the iterative nature of implementation, with sustainment portrayed as a quality improvement process where previous experiences provide feedback to the system between cycles of evaluation as a form of CQI. By extending structural models to include dynamic feedback, sustainment can be directly modelled, providing a framework to facilitate ongoing practice improvement and assess implementation success in complex service systems.

### Limitations of the current study

While our approach has a number of advantages, as discussed above, it also has some limitations. As outlined in the previous paragraphs, the multilevel decision juncture model is informed by scientific theories and existing evidence. Such models assume completeness, with no causal pathways unaccounted for. In other words, the absence of a path between two variables in a DAG implies the absence of a causal relationship [21]. Much of the criticism of non-experimental studies is motivated by such assumptions since the number of confounding paths can be substantial in social and health research and ignorability or unconfoundedness can generally not be tested [57]. However, we argue that this critique does not by itself invalidate the significant contribution that structural causal models can make to implementation research. With a focus on CQI and sustainment in complex systems, it is important to identify not only which implementation strategies may be effective but also how these strategies work within the system. Hence, causal explanation [58] is the key to identification of leverage points and feedback loops in the system. This is the strength of theory-driven causal models.

It is also important to note that the described approach does not depend on a particular study design. Tasks 1 and 2 are independent of the statistical approach to estimate parameters but are concerned with the formulation of a model and theoretical identification of effects [33]. Only during Task 3 will the study design matter. As such, the multilevel decision juncture model can be applied to inform both experimental and non-experimental studies. With a focus on CQI and sustainment, we have restricted our discussion to routine data collection settings based on observational data. Consequently, this approach requires careful validation of the models employed for analysis, including theoretical justification and sensitivity testing [48,59,60], which should be conducted in any rigorous scientific investigation.

Another limitation of the current study is the assumed availability of measures for all variables in the model. Despite an abundance of measurement instruments [56], few studies have focused on measuring implementation in practice settings. Theoretical models facilitate data collection by highlighting confounding factors that should be measured. Recent efforts in implementation research focus on the development of flexible data collection platforms that facilitate the use of structural models in real-world practice environments [55].

In situations where not all required data is available to block confounding pathways, alternative techniques such as instrumental variable estimation [38,61] must be used during Task 3. Theory-based models can justify the use of these techniques by explicating underlying assumptions.

Addressing practitioners and researchers interested in the implementation of evidence-based practices alike, the current study has also postponed a detailed treatment of the analysis of dynamic models. However, the applied example in this study can be extended to capture dynamic processes, including feedback of implementation and intervention outcomes on latter implementation cycles. In real-world implementation contexts, dynamic processes are of particular interest as they are at the core of sustainment and CQI efforts. However, treatment of these analytical extensions is technically involved and beyond the scope of this study and we refer the interested reader to the relevant literature [21,31,62].

## Conclusions

Implementation is ultimately an optimization process in the use of interventions to maximize patient outcomes. The systems decision perspective described in this paper provides a starting point towards retrieving parameters of causal mechanisms throughout the process of implementation as an integrated system. At any phase or stage of the implementation process, parameters can be estimated, with causal mechanisms modelled and studied to learn about decision making processes in the system. The effects of implementation on patient outcomes are clearly specified through an explicitly described causal chain, with pathways effected by branches based on choices at decision junctures. Such an approach focuses on the pathways through which policies and strategies work in the system, and this has the potential to speed up the translation of research knowledge into practice. From such a perspective, causal explanation [58] is of central interest and this is where decision-based structural models will be indispensable for future implementation research in complex systems of healthcare.

## Supporting information

S1 Appendix Supplementary material. (PDF)
