# Supplementary material for: Decision-based models of the implementation of interventions in systems of healthcare: Implementation outcomes and intervention effectiveness in complex service environments
Source: PLoS One. 2019 Oct 17;14(10):e0223129. doi: 10.1371/journal.pone.0223129 (PMC6797080; doi:10.1371/journal.pone.0223129)
Supplement: S1 Appendix — (PDF) [file pone.0223129.s001.pdf]

Decision-based models of the implementation of interventions in systems of health care: Implementation outcomes and intervention effectiveness in complex service environments - Supplementary Material

Arno Parolini* Wei Wu Tan* Aron Shlonsky±

In this supplementary file, we explain the underlying concepts and assumptions of the structural decision-based implementation approach. We discuss how decisions in the implementation system are related to structural choice models and provide additional information on the data generation and analysis of the hypothetical case study in the main text. The aim is to provide sufficiently rigorous background information to demonstrate the validity and benefits of the structural approach to implementation science.

While there are different frameworks for causal inference, we focus on the Pearl Causal Model (PCM) in this supplement due to its wide application. However, it is important to note that the treatment here is for introductory purposes and the interested reader is referred to the references cited throughout the article for a thorough discourse of the topic.

We structure the supplement as follows: After the fundamental conceptual approach underlying the decision-based implementation system has been outlined in Section A.1, we provide a general description of directed acyclic graphs (DAG) in Section A.2. Section A.3 provides more detail on the hypothetical case example, and formal expressions of the causal effects, making use of the concepts outlined in Section A.1 and the theorems from the causal inference literature. Section A.4 includes the Stata code for running the Monte-Carlo simulations.

---

*University of Melbourne

± Monash University

A.1 Decisions and Structural Causal Models

In this section, we discuss the decision-based causal approach in greater detail. The structural approach sits at the center of the decision-based implementation system and therefore it is important to understand the underlying concepts of structural causal models and choice theory. However, the discussion here provides only a general overview of the topic as a thorough treatment cannot be covered in a single manuscript and we refer the interested reader to the references cited throughout this online supplement.

Before we describe implementation decisions as choice processes, we begin by defining a structural equation:

$$y = f(X, u) \qquad (1)$$

Such an equation is deemed structural if the relationship represented by the function $f(X, u)$ is causal, i.e., if the outcome 'y' is caused by the set of explanatory variables 'X' with an error term 'u' representing all other unobserved causes of 'y' that are independent of variables in 'X'. This follows Goldberger [1], who defined structural equations as "stochastic models in which each equation represents a causal link, rather than a mere association" (p.979). In equation (1), stochasticity refers to the presence of unobserved causes represented by 'u'.

To integrate the structural approach within a decision framework, we must examine the behavioral process leading to decision makers' observed choices [2] and thus will take some time to explain this process as it is fundamental for understanding our approach.

We assume that a person acting within the implementation system can choose from a finite set of available options. For example, a manager in an organization may choose between several implementation strategies. Two crucial but not very restrictive assumptions are that the options are mutually exclusive (i.e. choosing one option means a decision maker cannot

choose an alternative) and that the set of options is complete, meaning that there are no valid options that are unaccounted for [2].

An observed choice made by an actor in the system is a result of a comparative process. A decision maker carefully weighs the benefits and costs of each alternative, which are not necessarily measured in monetary units, and compares the net benefits of available options to come to a conclusion. In the academic literature on discrete choice modeling, this net benefit is referred to as utility. Unfortunately, utilities are generally unobserved and researchers only have information on the observed choices. For example, assuming two alternatives, the decision rule guiding the choice of a particular option in a given choice situation consists of two parts: (1) the relationship between the utilities and factors influencing the decision, and (2) the relationship between the utilities and the observed choice. Assuming a simple linear relationship between benefits and costs for illustration purposes, the two components of a particular decision maker's choice option (indexed by *i*) in a given choice situation (indexed by *k*) can be expressed as follows:

$$NB_{i,k} = B_{i,k} - C_{i,k}\,, \quad i = \{1,2\}, k = \{1, \ldots, K\} \tag{2}$$

$$L_k = \begin{cases} 1 & if\ NB_{i,k} > NB_{j,k}\ , \forall i \neq j\ and\ \ i,j \in \{1,2\}, k = \{1, \ldots, K\} \\ 0 & otherwise \end{cases} \tag{3}$$

Equation (2) states that the net benefit ($NB_{i,k}$) of an option is the difference between its resulted benefits ($B_{i,k}$) minus its incurred costs ($C_{i,k}$). As mentioned above, benefits and costs are not necessarily measured in monetary terms but can also include individual or alternative specific characteristics, allocated time, and non-tangible elements such as stigma. Equation (3) relates the concept of unobserved utilities to actually observed choices by defining a discrete outcome variable (L). For now, let us assume the outcome is a categorical variable with two classes represented by 0 and 1. Only the outcome variable of the option with the highest net utility will have the value of 1 while those of all other options will have the value

of 0. The decision rule in equation (3) states that an actor will choose the option with the highest net utility instead of the other alternatives.

If researchers had measurements available for all the factors influencing actors' decisions, the relationships between the observed choices and their inputs would be deterministic. However, this is unlikely and therefore the relationship represented by equation (1) involves uncertainty represented by an error term. Accounting for this uncertainty, equation (2) becomes:

$$NB_{i,k} = B_{i,k} + U_{i,k}^{B} - C_{i,k} - U_{i,k}^{C}, \quad i = \{1,2\}, k = \{1, \dots, K\} \qquad (4)$$

Where $U_{i,k}^{B}$ represents unobserved benefits and $U_{i,k}^{C}$ denotes unobserved costs of a particular alternative in a particular choice situation (k).

Based on their knowledge or other evidence, researchers can make probabilistic statements about actors' choice processes which express the likelihood that a decision maker will pick a particular option given the observed circumstances. Many different models have been suggested in the academic literature on discrete choices [2,3].

In addition to actors' decisions, we are interested in the effects of interventions as part of our implementation system. Again, we can formulate the relationship between the outcome of interest, a treatment variable and other causes of the outcome in the form of equation (1). The exact specification will again depend on researchers' assumptions based on knowledge and existing evidence.

A structural causal model (see Definition 7.1.1 of Pearl [4]) consists of a set of structural equations. Each equation represents a causal relationship between an outcome, or dependent variable, and the explanatory variables of interest, which are often denoted as 'parents' of a particular outcome variable [4]. At the core of structural causal models or structural systems rests the assumption that the equations represent an autonomous set of mechanisms [5]. The

assumption of autonomy implies that the relationships between an outcome variable *‘y’* and its parents ‘*X*’ in each of the equations are unaffected by how the values of ‘*X*’ are determined [6]. For example, the relationship between blood sugar level of a diabetes patient (*‘y’*) and insulin injection (a variable in set *‘X‘*) will be the same whether insulin injection (the value of a particular *‘X’*) was determined through external manipulation (e.g. a controlled experiment) or another mechanism observed in the real world.

A.2 A Brief Introduction to Directed Acyclic Graphs

Directed acyclic graphs are a special form of causal graphs illustrating the relationships between elements of a system. As the concept of DAGs plays a central role in this study, we provide an easily accessible but informal description of the most essential concepts. More thorough treatments of causal graphs and their properties are provided by White and Lu [7] or Pearl [4].

A graph *G* consists of a set of nodes (or vertices) and edges that connect nodes with each other [7]. For the purpose of this study we assume that nodes in graphs represent random variables, as is standard in causal analysis. Variables included in the graph can be measured or unmeasured.

The edges in a graph represent the relationship between random variables. Directed relationships are represented by single headed arrows pointing from an initial node (direct cause) to a terminal node (outcome). As stated in section A.1, direct causes are also referred to as parents. Bi-directional relationships in a graph are represented by double headed arrows. Relationships involving at least one unmeasured variable are denoted by dashed arrows while relationships between measured variables are usually symbolized by solid arrows [4]. Variables in the graph that have no parents (i.e., no edge leading into them) are exogenous

variables in the system (or roots [7]). All other variable are endogenous variables determined within the system.

One or more edges form a path between an initial node and a terminal node. A directed graph is a graph that consists only of directed edges [7]. On a particular path, an edge connects with another edge through a common node to form a sequence. If all edges of a path are directed and each terminal node is also the initial node of the next variable, then a path is a directed path [7]. For example, in the case of a linear path, this would mean that all edges point in the same direction. Finally, if the initial node of a path is also the final node on the path, then the path is a cycle (e.g., a feedback loop). A directed acyclic graph is a directed graph without cycles.

An important concept within the causal inference literature that is directly related to DAGs are back-door paths [4], which indicate the presence of confounding variables that can distort the identification of effect of interest between two variables.

Related to the back-door path is the back-door criterion, which refers to a set of variables with certain conditions that can be used to block all back-door paths [4].

A.3 Hypothetical Case Example of Implementing a Treatment Program for Anxiety Disorders in Children and Adolescents based on Cognitive Behavioral Therapy

Assumptions

In this section, we provide a more detailed treatment of the hypothetical case study described in the main article. We state several assumptions that we make in the example, mainly to aide with conveying the approach described in this study.

This section is based on the scenario illustrated in Fig 3 of the main article and expands on the Directed Acyclic Graph (DAG) presented in Fig 4. The expanded DAG is shown in Fig A1 below, with all variables the same as those in Fig 4 in the main articles except variables

designated as U_i, which are unobserved causal variables. As outlined in the main article, causal graphs explicate all assumptions about causal relationships within a system [4] and causal interpretation can be justified under conditions such as available controls for direct causes or ignorability, as defined in the causal inference literature [4,7,8].

Before we proceed to the analysis of the model, we discuss the assumptions made in the hypothetical case example of this study in more detail:

1. We restrict our attention to the first time a patient is recorded in the system within the observation period and exclude patients' simultaneous engagement with multiple organizations. This assumption simplifies the example and is closely related to the second assumption below. Furthermore, this assumption is in line with most randomized controlled trials, making our approach comparable and more familiar to readers. Relaxation of this assumption leads to a dynamic model where treatment levels or intensity depends on previous treatment and outcomes [4,9].

2. We assume that there is no feedback of implementation or treatment outcomes to clinicians or organizations, i.e. that their information set is constant for the observation period. Such an assumption is reasonable for an early implementation setting, assuming that the intervention is delivered by outside facilitators or that the assignment period is short, relative to the duration of the program. This assumption, in combination with assumption 1, allow us to specify this model as a recursive system (i.e., a system without feedback). Relaxing this assumption would require us to specify a state-dependent dynamic model where decision makers take previous outcomes into consideration as would be the case for sustainment and CQI decision cycles. However, treatment of state-dependent dynamic models is beyond the scope of this study.

3. Furthermore, we assume that patients' outcomes are independent (e.g., no social interaction), that the implementation strategy does not alter the treatment regimen (i.e., there are no hidden treatment variations), and that treatment in this case study is defined as a binary variable taking values 0 or 1.

4. All observed variables are measured without error (e.g. X_2 is a perfect proxy for the latent construct of perceived leadership). This assumption simplifies the example significantly but can be relaxed by the introduction of measurement models [9]. Such cases would require a different graphical representation that considers latent variables such as multiple indicators, multiple causes (MIMIC) models [1]. Again, treating such examples is outside the scope of this article.

In relation to treatment effects, these assumptions justify the stable unit treatment value assumption (SUTVA) that is often implicitly assumed in the experimental literature [8,10]. It is important to note that in the presented example, measured variables are available to block back-door paths in order to identify causal effects [4]. In a nonparametric framework, this situation is equivalent to matching on covariates [6].

Identification of causal effects

To illustrate the structural approach, we will formally derive effects described by three research questions: (1) Did the enhanced implementation option increase perceived feasibility and appropriateness among clinicians? (2) Did an increase in perceived feasibility and appropriateness of the intervention among practitioners increase the probability of treatment assignment for patients? (3) Did Cognitive Behavioral Therapy (CBT) have a positive effect on patients' outcomes? These questions are in line with the two components of hybrid designs [11] and are also described in the main article. However, the structural causal model described here investigates the causal links between these elements across different levels of

the system and these are not formally captured by hybrid designs. The benefit of structural systems is that they enable researchers to answer more complex questions that go beyond what we can possibly learn from randomized controlled studies, including the investigation of hypothetical interventions, causal paths, and mediation effects [12].

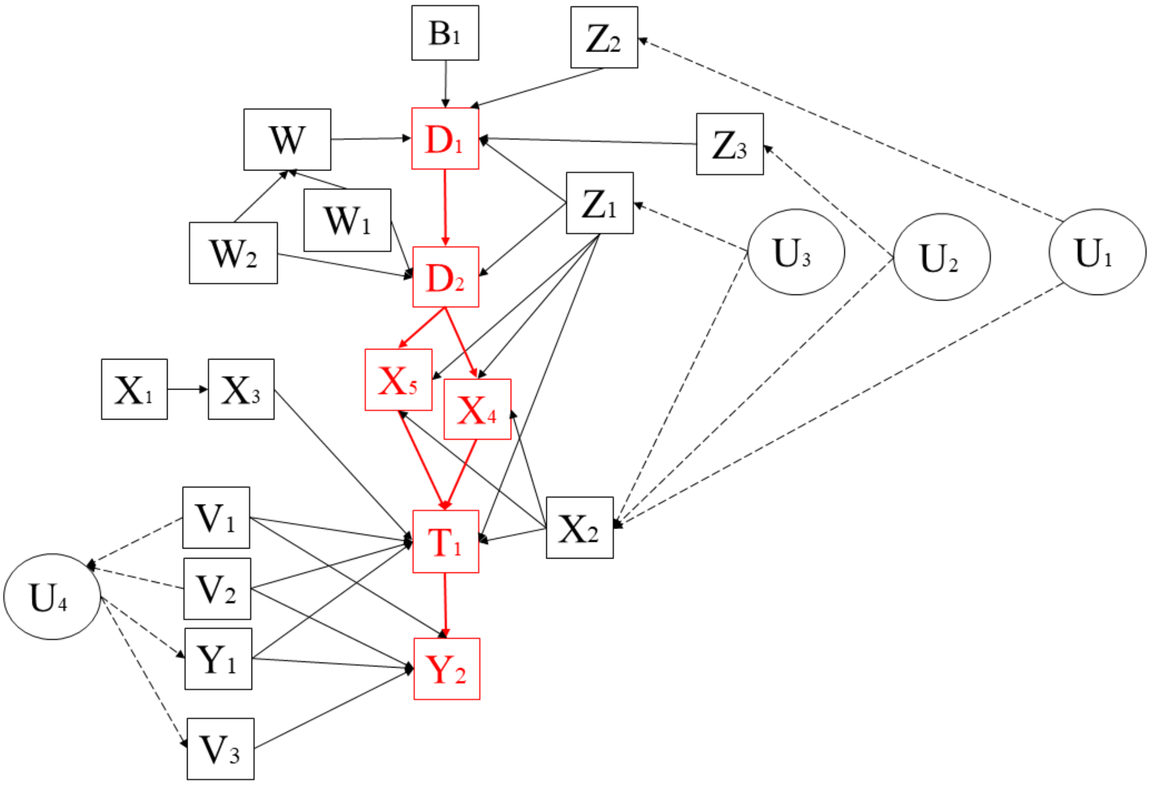

Fig A1. Directed Acyclic Graph of the structural model for CBT for anxiety disorders in children and adolescents [1]

[1] Observed variables are in squares while unobserved (sets of) variables are in circles. Variables D_1, D_2, and T_1 represent the overall decision to implement, the choice of implementation strategy and the decision to apply treatment to patient respectively. Dashed arrows emanating from the unobserved errors (U_i) towards two observed variables represent unobserved correlations between the variables (i.e., correlated errors between two variables, usually referred to as confounding). Each node can be viewed as having an unobserved error as input. Following conventions in the literature unobserved errors affecting only one node are not shown in the graph [4].

For the purpose of this section, we extend the DAG from Fig 4 in the main article to explicate the unobserved causal variables. The inclusion of additional variables compared to Fig 4 aids the selection of covariates with the aim of increasing efficiency of estimates [7].

The structure described in Fig A1 can be justified by the temporal ordering of the decisions over the implementation process. However, as outlined above, we do not discuss models where organizations' and practitioners' decisions are contingent on previous implementation cycles and/or patient outcomes as such examples are significantly more involved and are beyond the scope of this study.

Since the DAG in Fig A1 represents a structural causal model it corresponds to the following set of structural equations:

$U_1 = f_{U1}(\varepsilon_{U1})$ $\quad$ $U_2 = f_{U2}(\varepsilon_{U2})$

$U_3 = f_{U3}(\varepsilon_{U3})$ $\quad$ $W_1 = f_{W1}(\varepsilon_{W1})$

$W_2 = f_{W2}(\varepsilon_{W2})$ $\quad$ $W = f_W(W_1, W_2, \varepsilon_W)$

$V_1 = f_{V1}(\varepsilon_{V1})$ $\quad$ $V_2 = f_{V2}(\varepsilon_{V2})$

$Y_1 = f_{Y1}(U_4, \varepsilon_{Y1})$ $\quad$ $V_3 = f_{V3}(U_4, \varepsilon_{V3})$

$Z_1 = f_{Z1}(U_3, \varepsilon_{Z1})$ $\quad$ $Z_2 = f_{Z2}(U_1, \varepsilon_{Z2})$

$Z_3 = f_{Z3}(U_2, \varepsilon_{Z3})$ $\quad$ $U_4 = f_{U4}(V_1, V_2, \varepsilon_{U4})$

$D_1 = f_{D1}(B_1, Z_1, Z_2, Z_3, W, \varepsilon_{D1})$ $\quad$ $D_2 = f_{D2}(D_1, Z_1, W_1, W_2, \varepsilon_{D2})$

$X_1 = f_{X1}(\varepsilon_{X1})$ $\quad$ $X_2 = f_{X2}(U_1, U_2, U_3, \varepsilon_{X2})$

$X_3 = f_{X3}(X_1, \varepsilon_{X3})$ $\quad$ $X_4 = f_{X4}(D_2, Z_1, X_2, \varepsilon_{X4})$

$X_5 = f_{X5}(D_2, Z_1, X_2, \varepsilon_{X5})$ $\quad$ $T_1 = f_{T1}(Z_1, X_2, X_4, X_5, V_1, V_2, Y_1, \varepsilon_{T1})$

$$Y_2 = f_{Y2}(T_1, V_1, V_2, V_3, Y_1, \varepsilon_{Y2})$$

In the equations above, the $f_i(\cdot)$ represent deterministic functions and ε_i represent population error terms. These are generally not equal to the residuals from regression models. It is important to note that the system of structural equations above does not put any restrictions on the functional form of the model. Given that Fig A1 is a DAG with the presence of unobserved variables $U \equiv \{U_1, U_2, U_3, U_4\}$, the causal model can be described as semi-Markovian [4].

Below, we will use graphical conditions to identify causal effects in Fig A1 before implementing the structural causal model within a Monte-Carlo simulation study described in section A.4. Alternatively, these effects can be derived using algebraic operations such as 'do-calculus' [4] or using hypothetical models and 'fixing' as described by Heckman and Pinto [6]. In the discussion that follows, we will treat all conditioning variables as discrete to enable the use of summation rather than integrals. This should facilitate the theoretical discussion of the identification of causal effects and is in line with the usual treatment in the literature [4,6].

Effect of implementation strategy (D_2) on perceived feasibility (X_4) and appropriateness (X_5) of the intervention:

The estimation of the effect of D_2 on X_4 and X_5 requires us to control for confounding as there exist unblocked back-door paths between D_2 and the two dependent variables [4]. Using the back-door criterion (Theorem 3.3.2 of Pearl [4]) allows us to block any confounding paths and the average treatment effect (ATE) can be estimated as:

$$E\left(x_k \middle| \hat{d}'_2\right) - E\left(x_k \middle| \hat{d}''_2\right) = E(x_k | d'_2, z_1, x_2) - E(x_k | d''_2, z_1, x_2), \; k \in \{4,5\} \quad (1)$$

Where d'_2 and d''_2 represent two distinct realizations of D_2 while the same variables with a hat symbol on top, i.e. $\hat{d}'_2$ and $\hat{d}''_2$ on the right hand of equation (1), indicate the fixing of variable D_2 to particular values of the variables. It is important to note that the expected values in equation (1) refer to pre-intervention probabilities [4]. Despite being direct causes of D_2, W_1 and W_2 should not be included as covariate as this decreases efficiency [7]. Furthermore, the fact that D_1 is binary can cause problems due to complete separation (i.e., the probability $P(do(D_2 = d'_2))$ may not be strictly positive as required for causal effect identifiability [4]) because D_1 leads to perfect prediction of treatment assignment in organizations without intervention adoption. However, following the studies by Heckman et al. [9] and Pearl et al. [13], we can investigate the effect of D_2 on T_1 conditional on organizations implementing the intervention (i.e., D_1 = 1). Researchers should generally be aware of overlap and support conditions [14]. Hence, we can estimate the average causal effect of implementation strategy on each implementation outcome as:

$$E\left(x_k \middle| \hat{d}'_2, D_1 = 1\right) - E\left(x_k \middle| \hat{d}''_2, D_1 = 1\right) \qquad (2)$$

$$= E(x_k|d'_2, z_1, x_2, D_1 = 1\ ) - E(x_k|d''_2, z_1, x_2, D_1 = 1), \qquad k \in \{4,5\}$$

Effect of implementation outcomes on treatment assignment (T_1):

As can be seen from Fig A1, there are several pathways leading from X_4 and X_5 to T_1. Since all direct causes of X_4 and X_5 are measured, we can identify the causal effect of implementation outcomes on T_1 by adjusting for these direct causes (Theorem 3.2.2 of Pearl [4]), assuming all conditioning variables are discrete to facilitate the presentation. The ATE of setting (or fixing) variable X_4 from value x to x+1 can then be derived by building the difference:

$$E(t_1|\hat{x}'_4) - E(t_1|\hat{x}''_4) = P(t_1|\hat{x}'_4) - P(t_1|\hat{x}''_4) \qquad (3)$$

$$= \sum_{pa_{x4}} P(t_1|x'_4, pa_{x4})P(pa_{x4}) - \sum_{pa_{x4}} P(t_1|x''_4, pa_{x4})P(pa_{x4})$$

Where x'_4 and x''_4 represent two distinct realizations of variable X_4 and pa_{x4} denotes the set of parent variables (direct causes) of X_4. Again, probabilities in equation (3) represent pre-intervention probabilities [4]. The second equation follows from the fact that T_1 is dichotomous. The conditioning on $D_1 = 1$ is implicit in equation (3) to simplify notation. Identification of the treatment effect of setting variable X_5 from value x to x+1 can be achieved in the same way to equation (3).

Furthermore, by the back-door criterion [4], it is also possible to identify the effect of X_4 on T_1 by controlling only for Z_1 and X_2 and X_5. Given that X_2 and Z_1 and X_5 are all direct causes of T_1, this model should be preferable from an efficiency perspective [7].

Assuming that all conditioning variables are discrete, we can estimate the average causal effect of a discrete change in perceived feasibility on treatment assignment as:

$$E(t_1|\hat{x}'_4) - E(t_1|\hat{x}''_4) = P(t_1|\hat{x}'_4) - P(t_1|\hat{x}''_4) \tag{4}$$

$$= \sum_{z_1, x_2, x_5} P(t_1|x'_4, z_1, x_2, x_5)P(z_1, x_2, x_5) - \sum_{z_1, x_2, x_5} P(t_1|x''_4, z_1, x_2, x_5)P(z_1, x_2, x_5)$$

It is important to note however, that the outcome variable T_1 in our model varies at patient level while D_2 is an organizational characteristic. Furthermore, the omission of variable X_3, a caseworker characteristic, from the model will cause level-1 error terms to be correlated within caseworkers. Hence, valid statistical inference will require controlling for clustering in the sample [15]. Again, the estimation of the effect of X_5 on T_1 follows the same approach as equation (4).

For increased efficiency, all variables that are direct causes of the dependent variable (X_3, V_1, V_2 and Y_1) should be considered as covariates [7]. This will also eliminate any unobserved

heterogeneity at cluster level in our model and, therefore, usual statistical inference should be valid.

These examples show a particular advantage of structural causal systems. The identification of causal effects can be achieved using different models, depending on data availability. The underlying assumptions are made explicit in the structural system.

In the previous paragraphs, we have discussed the incremental effects of discrete changes in the implementation outcomes measures. In the econometric literature, causal effects of continuous variables are often interpreted as average marginal effects (AME), which are represented by the first partial derivate of the conditional expectation for each individual with respect to the covariate of interest, holding all other covariates constant (and implicitly conditioning on $D_1 = 1$) [16]:

$$ME_{x_k,i} = \frac{\partial E(y_i|x_{ik}, pa_{xk,i})}{\partial x_{ik}},\ i = \{1,2,\dots,N\}, k \in \{4,5\} \qquad (5)$$

The average marginal effect can then be consistently estimated by the sample average of marginal effects [16].

$$AME_{x_k} = \frac{1}{N}\sum_{i=1}^{N} ME_{x_k,i}\,,\ i = \{1,2,\dots,N\}, k \in \{4,5\} \qquad (6)$$

Again, more efficient estimates of the AMEs for implementation outcomes can be achieved by controlling for parent variables of the dependent variable (T_1), which in our example satisfies the back-door criterion [4], thus eliminating confounding.

In linear models, AMEs are equal to the estimated coefficients but the two can differ significantly in nonlinear models [16]. In section A.4, we use AMEs to estimate the causal effects of X_4 and X_5 and the approach is demonstrated in the do-file code. Having a broad audience in mind, we refrain from discussing marginal effects in more detail and refer interested readers to more advanced texts [16,17].

Effect of treatment (T_1) on parenting outcome (Y_2):

Again, assuming that the researcher has access to the measures of the observed variables in Fig A1, the causal effect of treatment on patients' parenting scores can be identified. By the back-door criterion [4], a nonparametric estimand of this average causal effect can be obtained by controlling for the set $PA_{T1} \equiv \{V_1, V_2, Y_1\}$. Similar to the previous section, we can restrict our analysis to organizations that have actually decided to implement the intervention (i.e., D_1 = 1) but we do not make this condition explicit in the expressions below to simplify notation.

$$E(y_2|\hat{t}'_1) - E(y_2|\hat{t}''_1) = E(y_2|t'_1, pa_{T1}) - E(y_2|t''_1, pa_{T1}) \qquad (5)$$

since T_1 is dichotomous and Y_2 is a continuous variable. This can be confirmed by assessing each of the thirteen paths between T_1 and Y_2 individually. In accordance with d-separation (Definition 1.2.3 of Pearl [4]) and Markov Compatibility (Definition 1.2.2 of Pearl [4]), the set PA_{T1} blocks all back-door paths from T_1 to Y_2 and therefore the two variables are d-separated in a graph where all arrows emanating from T_1 are deleted.

Two things are important to note at this point. First, variable V_3 should also be included for efficiency. Second, variable X_2 should not be included as it would decrease the accuracy of the estimate.

A.4 Monte-Carlo Simulation of the Hypothetical Example

The data generating process (DGP) described in this section is directly based on the DAG illustrated in Fig A1. To reduce the complexity of the simulation, we have not modelled the decision node D_1 (the decision to implement), which introduces the implicit condition D_1 = 1 as discussed in the previous section. Where available, values for regressors are guided by descriptive statistics published in meta-analyses of cognitive behavioral therapy in child and adolescent patient populations [18–20] as well as studies published in the implementation

science literature [21]. The introduction of a complex multilevel data structure and realistic values for covariates in the model is based on the aim to demonstrate the use of decision-based structural models in real-world contexts. Several authors have emphasized the need to conduct Monte-Carlo simulations using data sets that approximate reality as much as possible [22,23]. However, to decrease computation time, we did not introduce complex functional forms for the covariate relationships in the model (i.e., interaction terms, polynomials or fractions).

Overall the DGP generates a hierarchical dataset with different organizations that employ multiple practitioners who work with several patients. This structure is typical for community mental health settings [24]. The sample size for the simulation was set to 6000 observations (i.e. patients) and we used cluster sizes that resemble real world allied health settings [24].

Simulations were undertaken in Stata SE 14.2 with the number of Monte-Carlo replications set to R=10000. The initial seed for the simulation was based on the 6-digit sales machine authorization codes at the bottom of three receipts for purchases from different stores at different dates. A random draw conducted in Excel dictated which numbers were assigned to the seed in a random order.

Given the structure of the system, all models in the simulations were fitted using multiple linear regressions and probit regressions and inference is based on the cluster-robust variance-covariance matrix where appropriate [15]. To measure the accuracy of the structural causal approach in our Monte-Carlo simulations, we assess the relative bias for each parameter, including the 95 per cent confidence interval of this statistic. For AME and ATE estimates, relative bias is measured relative to the sample AME and ATE based on the true parameters. For linear models, this overlaps with the true parameters defined in the DGP while for probit models we calculated the AME as demonstrated in equations (5) and (6) for

each sample [16]. Relative bias for the coefficients was calculated relative to the true parameters defined in the DGP. Confidence intervals for these measures are based on the empirical standard error of the statistic [22,23].

In addition, we present the coverage rates for estimated coefficients in the sections below. Coverage reflects the number of times that the true value of the parameter was situated within the 95 per cent confidence interval of the estimate produced by the software. These confidence intervals were based on a normal approximation for probit coefficients [23] and the t distribution was used for linear regressions. Coverage rates should ideally be very close to 95 per cent for a 95 per cent confidence interval. However, non-coverage does not necessarily imply a bias in the estimated standard errors as this statistic is affected by the bias in parameter estimates, the bias in estimated standard errors and the distribution of parameter estimates [23].

As a final measure of performance of the model, we will assess the root mean squared error (RMSE) for each estimated coefficient. Similar to the relative bias statistic, this measure is based on the empirical standard error [22]. However, the standardized RMSE is a complete measure of accuracy as it spans both dimensions, bias and variability of the estimate [22,23]. Moreover, the RMSE is on the same scale as the original parameter [22,23]. For the analysis here, we have normalized the RMSE to represent the RMSE as percentage of the true value of the parameter.

Relative bias and coverage of parameter estimates

In Fig 5 of the main article, relative bias for the average marginal effects and the average treatment effects was assessed based on the true marginal effects calculated in each sample. For linear models, these estimates are equal to the regression coefficients, as explained above. However, for nonlinear models, the AME and ATE can differ substantially from the

estimated coefficients. In general, nonlinear estimators are not unbiased in finite samples but estimates approach true values as the sample sizes increases towards infinity [16]. Reduced models (i.e., population averaged models based on the minimum conditioning set to block back-doors) generally do not yield consistent estimates for the regression coefficients despite providing consistent estimates of the AME and ATE. However, for fully specified models, we can assess consistency of the coefficient estimates and whether the inference based on these estimates is accurate. This is a result of the chosen functional specifications of causal relationships in the DGP.

The relative bias and 95 per cent confidence intervals are presented in Fig A2. For completeness, we also included the estimates for linear models, which are identical to the AME and ATE presented in Fig 5 of the main text. The results reveal that the average relative bias for each coefficient is negligible.

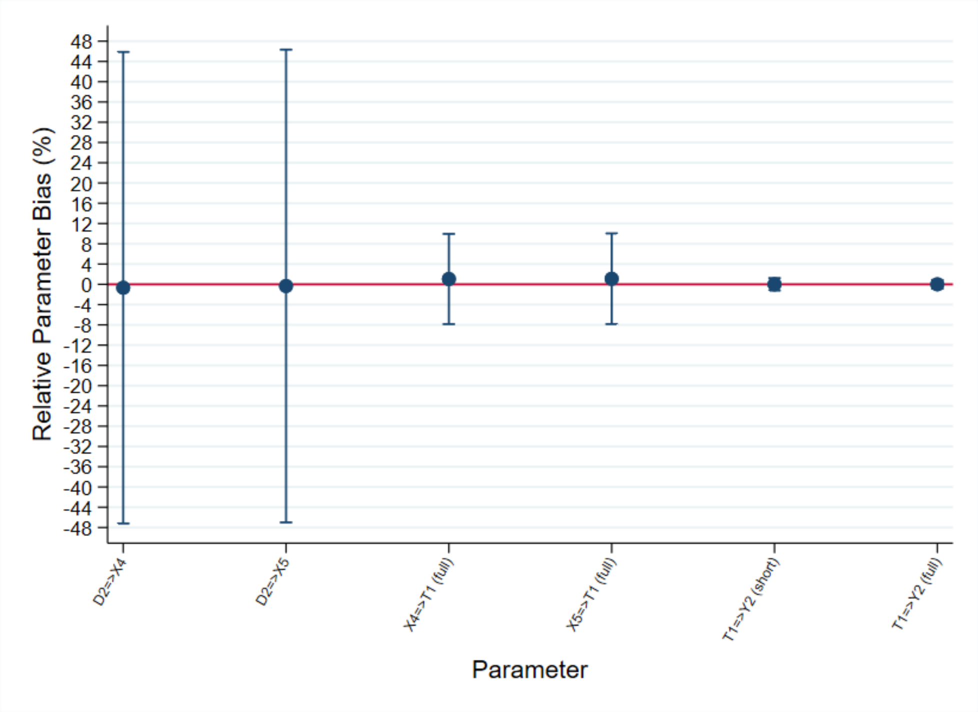

Fig A2. Relative parameter bias from Monte-Carlo simulations

Coefficients from probit regressions exhibit larger relative bias than linear models but these are still small in magnitude for perceived feasibility (X_4=>T_1 *(full)*; 1.05 per cent) as well as appropriateness (X_5=>T_1 *(full)*; 1.1 per cent). Moreover, all confidence intervals include zero.

Additional simulations using larger sample sizes (not shown) also revealed that the relative bias in probit estimates disappears as sample sizes at level-1 become large, which corroborates the finding that the estimates are not unbiased but consistent. As the sample sizes go to infinity, the asymptotic distribution therefore approximates the sample distribution and any bias in the estimated coefficients decreases towards zero.

The RMSE for each of the estimated coefficients corroborates the previous results. In Fig A3, the RMSE is normalized by the true parameter as defined in the DGP [23] and multiplied by 100 to express the statistic in per cent. Again, estimates for the effects of D_2 on X_4 and X_5 are relatively inaccurate. However, this is due to the particular DGP for these implementation outcome measures in the simulation rather than evidence of the inefficiency of the approach itself, which can be seen in the Stata do-file code. The standardized RMSE for other coefficient estimates are within the five per cent range of the true value. Overall, the results presented here show that structural causal models based on theory are well suited to estimate the effects of implementation strategies and outcomes on each other, as well as on patient outcomes.

In addition to consistency of estimates, it is important to investigate whether statistical inference based on these estimates is valid. We therefore assess the coverage rate for each coefficient to see whether the empirical confidence intervals (using an approximation to the normal distribution for probit estimates and t distribution for linear regression coefficients) are close to the nominal confidence level of 95 per cent.

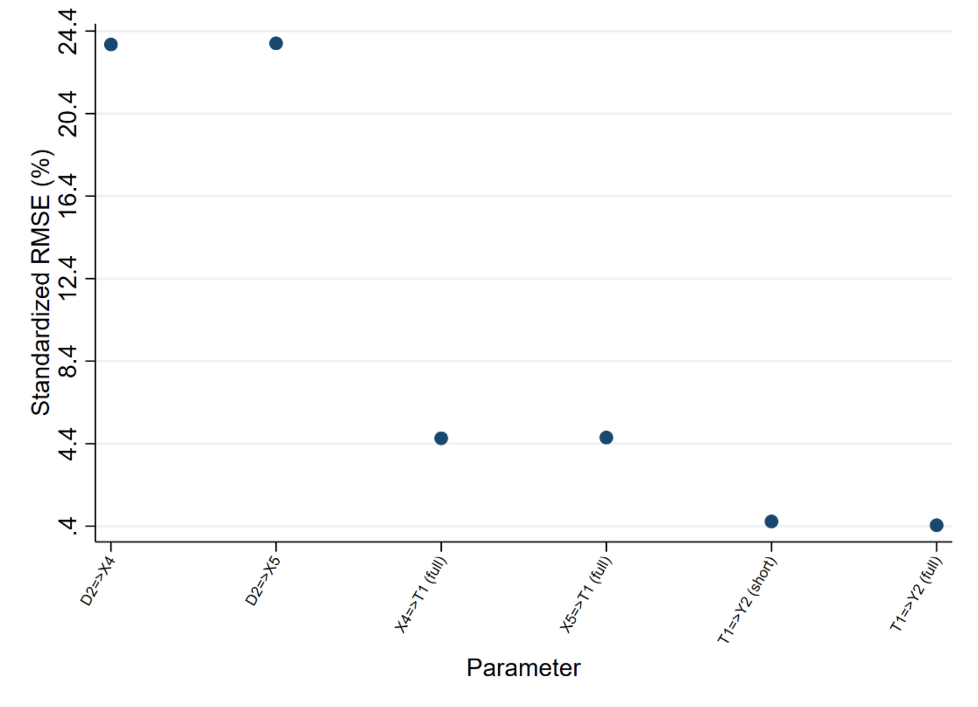

Fig A3. Standardized RMSE in per cent of true parameter

Fig A4 shows the coverage rates for each coefficient. The two horizontal lines in the graph represent the simulation interval, which accounts for the simulation error in coverage rates [22]. All coverage rates are very close to the 95 per cent nominal level, which indicates that statistical inference is generally valid.

However, the coverage rates for the effect D_2 on X_4 and X_5 appear to be outside the simulation interval, indicating that hypotheses tests based on this statistic may be subject to slight over-rejection and therefore slightly inflated Type-1 error. As pointed out earlier, coverage rates reflect a combination of factors including variability in estimates, bias in standard errors and distribution assumptions for estimates. Given that the variable D_2 is dichotomous and varies only at organizational level, the observed non-coverage is mainly caused by the large variation in estimates due to the small number of organizations in the data set. In fact, increasing the number of organizations to 30 already results in coverage rates for these estimates well within the simulation interval, as confirmed by sensitivity analyses shown in Fig A5.

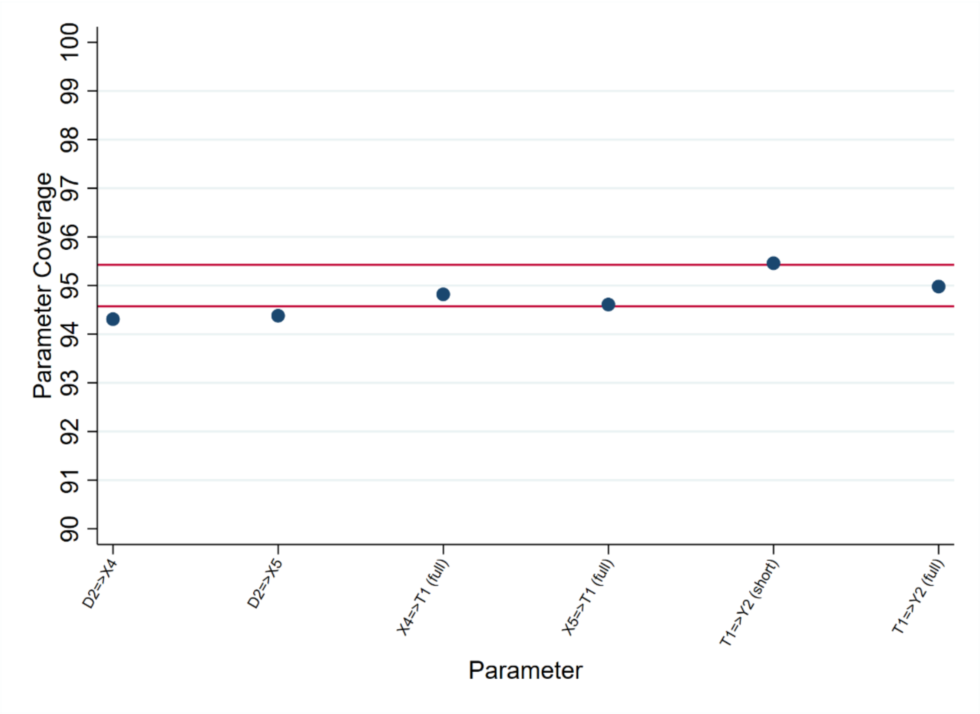

Fig A4. Coverage rates for true parameters and simulation interval

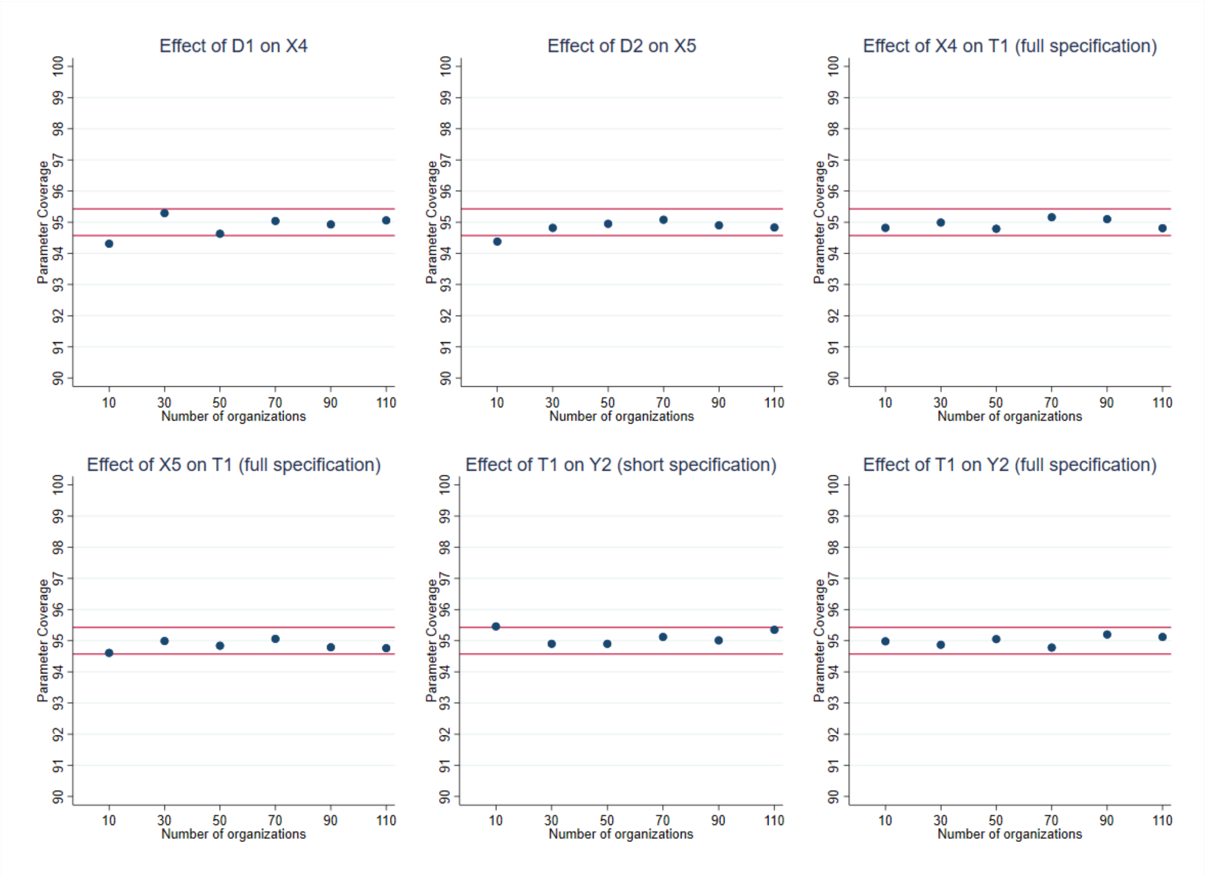

Fig A5. Coverage rates by number of organizations in the sample

Auxiliary sensitivity analyses are presented in Fig A6 and Fig A7 where we demonstrate the behavior of estimates as the sample size increases through the addition of further organizations from 10 to 110 in steps of 20.

As expected the relative bias in average marginal effects and treatment effects decreases as the number of organizations increases. Furthermore, given the structure of the system, the standardized RMSE is inversely related to the number of organizations in the sample. Hence, as sample size increases, estimates and statistical inference become more accurate. While our simulation results showed preferable properties as sample size increases, we decided to set the number of organizations to 10 for our main analyses to reflect more realistic conditions observed in allied health settings. This decision is justified by the fact that the deviation in the empirical confidence intervals from the nominal level of 95 per cent is very small. For the effect of D_2 on X_4, this deviation is only 0.69 percentage points and for the effect on X_5, the deviation is 0.62 percentage points. This amounts to an overall bias of 0.73 per cent and 0.65 per cent respectively and these biases include the simulation error.

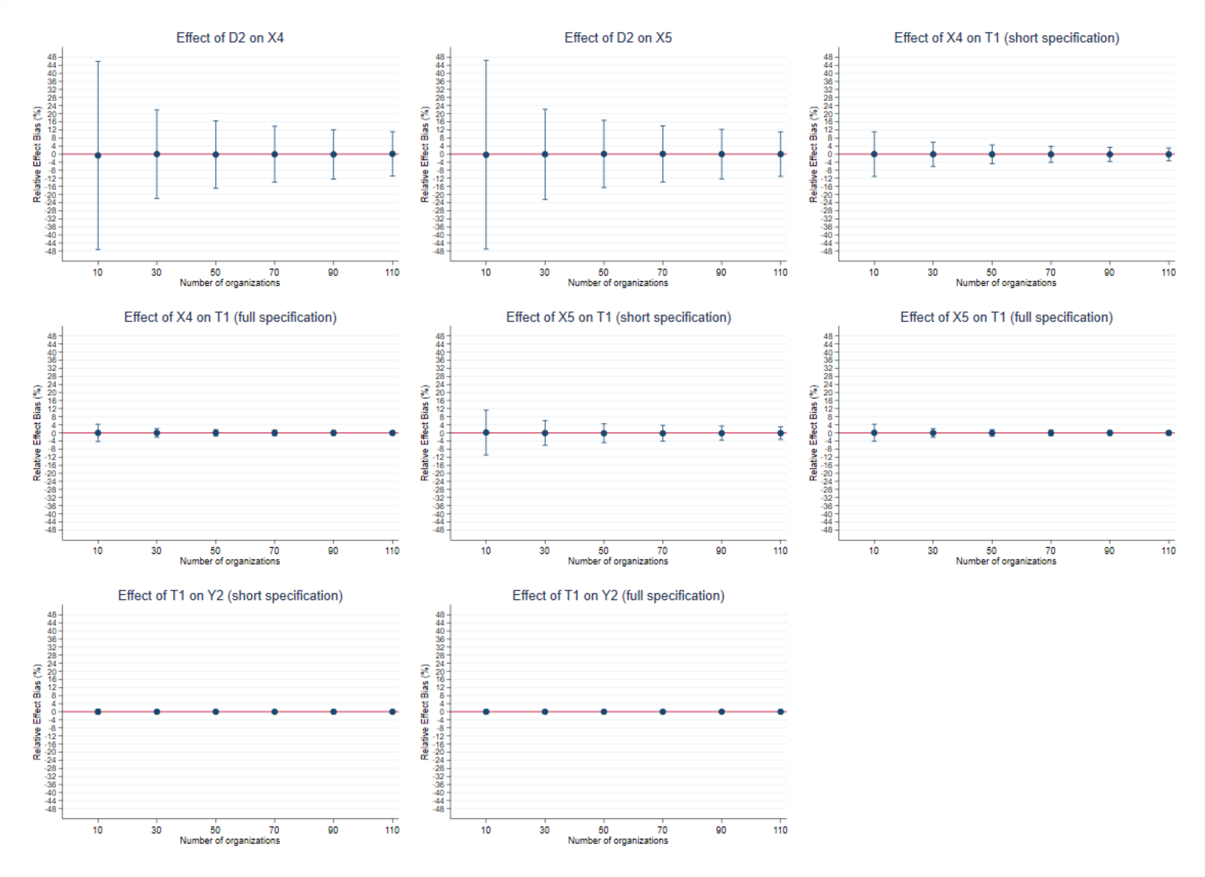

Fig A6. Relative bias in AME/ATE by number of organizations in the sample

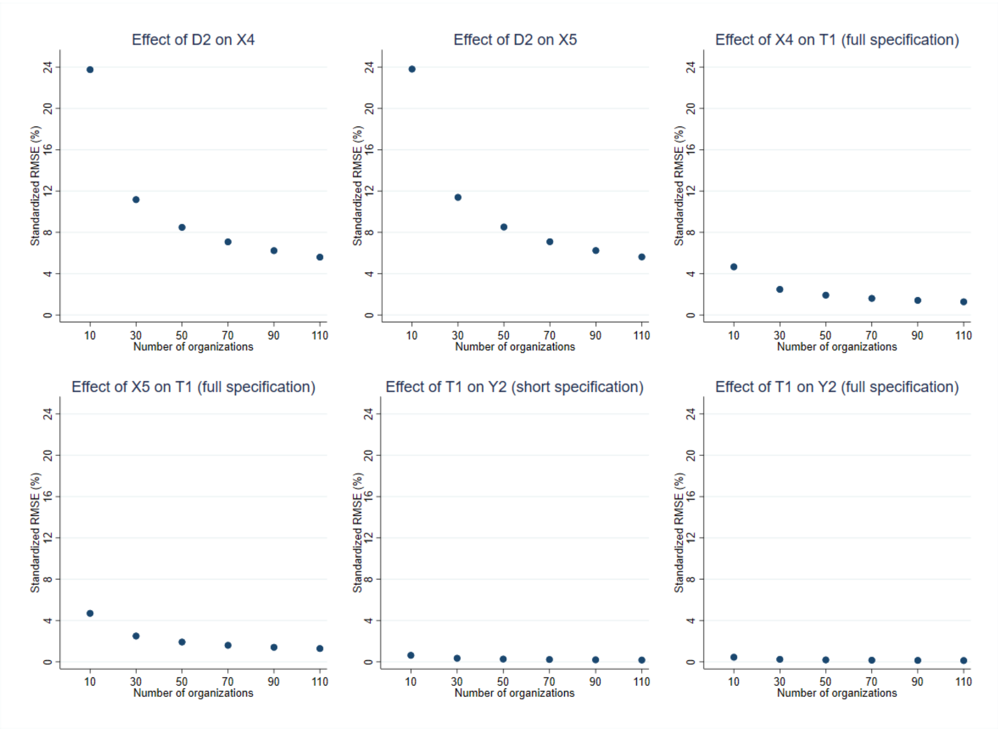

Fig A7. Standardized RMSE by number of organizations in the sample

Stata code to conduct simulation

```
capture log close
set more off
set matsize 5000
clear all
macro drop _all

************************************
**** Model parameters for MC sim ****
************************************

set seed 623504

global numsims = 10000          //number of repetitions
global nc = 30                  //patients per worker
global nw = 20                  //workers per organisation
global c = 10                   //Number of organisations or clusters

capture program drop struc_sim
program strucsim
	version 14
	tempname struc
	postfile `struc' ad2 at1 tme_feas1 tme_ap1 lin_fd2 sel_fd2 d_lf lin_apd2 sel_apd2 ///
d_la probit_feas sep_feas probit_ap sep_ap ame_feas_full se_feas_full ///
	ame_ap_full se_ap_full ame_feas_gee se_feas_gee ame_ap_gee se_ap_gee atate ///
b_t1 se_t1 df_r b_t1e se_t1e df_re using results_new_14, replace
	quietly {
		forvalues i = 1/$numsims {
			drop _all

*** True parameters for selection and outcome equations ***

*********************************
**** Generating true data set ****
*********************************

********** Generating hierarchies ***********

	set obs $c
	gen org_id = _n

*********** Organisational-level variables ************
	gen u1 = rnormal()
	gen u2 = rnormal()
	gen u3 = rnormal()

	gen ionet = runiform(0.2,0.8) + u3
	gen ostruc = 6 + u2 + rnormal()
	gen mstyle = 6 + u1 + rnormal()
```

```
************ Intervention-specific variables ***********
	gen sc1 = rnormal(20,1)
	gen sc2 = 20 + (1 + rchi2(3))
	gen m_sc = (sc1 + sc2)/2

******** Intercepts for clustered data, implementation leadership ********
	gen ics_int = rnormal(2.42,0.364)

******** Generating organisational utilities and decisions ***********

	* Relative utilities from each alternative *
	gen su = 5 + 10*ionet + 6*mstyle - (sc2 + sc1) + rnormal(0,2)
	gen d2 = su > 0
	sum d2
	scalar ad2 = r(mean)

************ expanding to clinicians *************

	expand $nw
	gen cw_id = _n

************ Clinician-level variables **************

* Practitioner age (based on [21]) assuming triangular distribution to ensure age range *
	local x = 21
	local z = 66
	local q = 3*36.5-(`x'+`z')
	gen runif1 = runiform()
	local F_c =  (`q'-`x')/(`z'-`x')
	gen age_cw = `x'+sqrt(runif1*(`z'-`x')*(`q'-`x')) if (runif1 < `F_c')
	replace age_cw = `z'-sqrt((1-runif1)*(`z'-`x')*(`z'-`q')) if (runif1 >= `F_c')
	replace age_cw = round(age_cw)

* Generating discrete exogenous variables *
	gen rand2 = runiform()
	gen gender_cw = rand2 <= 0.7

	* Tenure within position*
	gen tenure = age_cw - 21 - rnormal(16)
	replace tenure = runiform(0,1) if (tenure < 0)

	* Generating Implementation Leadership Scale (based on [21] (p.5))*
	gen ics = ics_int + u3 + u2 + u1 + rnormal(0,0.89)
	replace ics = 0 if ics < 0
	replace ics = 4 if ics > 4

	* Feasibility of Intervention *
	gen feas = 3 + 2*d2 + 0.3*ics + 0.5*ionet + 2*rnormal()

	* Appropriateness of Intervention *
```

```
	gen ap = 3 + 2*d2 + 0.3*ics + 0.5*ionet + 2*rnormal()

************* Expanding to patients ***********
	expand $nc
	gen id = _n
	bys cw_id: gen tagw = _n==1

************ Patient-level variables  **************

	* Patient age (based on [20]) assuming triangular distribution to ensure age *
	local r = 6
	local t = 18
	local l = 3*9.7-(`r'+`t')
	gen runif1b = runiform()
	local F_c2 =  (`l'-`r')/(`t'-`r')
	gen age_cg = `r'+sqrt(runif1b*(`t'-`r')*(`l'-`r')) if (runif1b < `F_c2')
	replace age_cg = `t'-sqrt((1-runif1b)*(`t'-`r')*(`t'-`l')) if (runif1b >= `F_c2')
	replace age_cg = round(age_cg)
	label var age_cg "Age patient"

	gen gender_cg = runiform() <= 0.7
	label var gender_cg "Gender caregiver"

	gen u4 = 0.1*age_cg + 0.2*gender_cg + rnormal()

	gen y1 = rnormal(30,2) + 0.1*age_cg + 0.2*gender_cg + u4
	label var y1 "Baseline outcome score"

	gen v3 = rnormal(50,10) + u4

*****************************************************************************
Investigation of implementation strategy on implementation outcomes and treatment decision
*****************************************************************************

/* Effect of D2 on Feasibility and Appropriateness  */

	reg feas d2 ics ionet if tagw, vce(robust)
	scalar lin_fd2 = _b[d2]
	scalar sel_fd2 = _se[d2]
	scalar d_lf = e(df_r)

	reg ap d2 ics ionet if tagw, vce(robust)
	scalar lin_apd2 = _b[d2]
	scalar sel_apd2 = _se[d2]
	scalar d_la = e(df_r)

/* Effect of D2 on Feasibility and Appropriateness  */

	gen t_mu = -19 + feas + ap - 0.5*age_cg + 2*gender_cg + 0.45*y1 + 0.6*ics -
0.1*tenure + 0.8*ionet
```

```
    gen e_t = rnormal()
    gen t_u = t_mu + e_t
    gen t1 = t_u > 0

    sum t1
    scalar at1 = r(mean)

*Calculating true marginal effects of implementation outcomes on treatment decision*
    gen tme_feas = normalden(t_mu)*1
    sum tme_feas, meanonly
    scalar tme_feas1 = r(mean)

    gen tme_ap = normalden(t_mu)*1
    sum tme_ap, meanonly
    scalar tme_ap1 = r(mean)

*Estimating the marginal effects from the sample*
    probit t1 feas ap age_cg gender_cg y1 ics tenure ionet
    scalar probit_feas = _b[feas]
    scalar sep_feas = _se[feas]
    scalar probit_ap = _b[ap]
    scalar sep_ap = _se[ap]
    margins, dydx(*)
    matrix B_io = r(b)
    matrix V_io = r(V)
    scalar ame_feas_full = B_io[1,1]
    scalar ame_ap_full = B_io[1,2]
    scalar var_feas_full = V_io[1,1]
    scalar se_feas_full = sqrt(var_feas_full)
    scalar var_ap_full = V_io[2,2]
    scalar se_ap_full = sqrt(var_ap_full)

    probit t1 feas ap ionet ics, vce(cluster cw_id)
    margins, dydx(*) vce(unconditional)
    matrix B_gee = r(b)
    matrix V_gee = r(V)
    scalar ame_feas_gee = B_gee[1,1]
    scalar ame_ap_gee = B_gee[1,2]
    scalar var_feas_gee = V_gee[1,1]
    scalar se_feas_gee = sqrt(var_feas_gee)
    scalar var_ap_gee = V_gee[2,2]
    scalar se_ap_gee = sqrt(var_ap_gee)

************************************************************************
/* Effect of T1 on Y2 */

            gen e = rnormal()
            gen yc = 1 + y1 + 0.4*age_cg + 0.2*gender_cg + 0.1*v3 + e
            gen yt = 8 + y1 + 0.4*age_cg + 0.2*gender_cg + 0.1*v3 + e
```

```
                    gen te_true = yt - yc
                    sum te_true
                    scalar atate = r(mean)
                    gen y_obs = yc + t1*(yt-yc)

                    reg y_obs i.t1 age_cg i.gender_cg y1
                    scalar b_t1 = _b[1.t1]
                    scalar se_t1 = _se[1.t1]
                    scalar df_r = e(df_r)

                    reg y_obs i.t1 age_cg i.gender_cg y1 v3
                    scalar b_t1e = _b[1.t1]
                    scalar se_t1e = _se[1.t1]
                    scalar df_re = e(df_r)

**********************************************************************
post `struc' (ad2) (at1) (tme_feas1) (tme_ap1) (lin_fd2) (sel_fd2) (d_lf) (lin_apd2) (sel_apd2)
(d_la) (probit_feas) (sep_feas) (probit_ap) (sep_ap) (ame_feas_full) (se_feas_full)
(ame_ap_full) (se_ap_full) (ame_feas_gee) (se_feas_gee) (ame_ap_gee) (se_ap_gee)  ///
(atate) (b_t1) (se_t1) (df_r) (b_t1e) (se_t1e) (df_re)
                }
        }
        postclose `struc'
end

*************************************************************************
******* Run simulation program *******
*************************************************************************

di "Time is: " c(current_time) " on " c(current_date)
set more off
strucsim

use results_new_14, clear
notes _dta: All variables in this file containing characteristics are averages from individual
cycles.

di "Time is: " c(current_time) " on " c(current_date)

gen run = _n

****************
*relative bias
****************
*Effect of implementation strategy on implementation outcomes

                gen rbias_feasd2 = 100*(lin_fd2/2 - 1)
                gen rbias_apd2 = 100*(lin_apd2/2 - 1)

*Effect of implementation outcomes on T1
```

```
		*Probit short
		gen rbias_feas_gee= 100*(ame_feas_gee/tme_feas1 - 1)
		gen rbias_ap_gee = 100*(ame_ap_gee/tme_ap1 - 1)	//relative bias treatment effect

		*Effect of full probit coefficients
		gen rbias_probit_feas = 100*(probit_feas/1 - 1)
		gen rbias_probit_ap = 100*(probit_ap/1 - 1)

		*Probit long
		gen rbias_feas_full = 100*(ame_feas_full/tme_feas1 - 1)
		gen rbias_ap_full = 100*(ame_ap_full/tme_ap1 - 1)	//relative bias treatment effect

* Effect of T1 on Y2

		*Reg short
		gen rbias_t1 = 100*(b_t1/atate - 1)

		*Reg long
		gen rbias_t1e = 100*(b_t1e/atate - 1)

******************
*calculating coverage
******************

*Effect of D2 on feas and ap
		gen lb_d2_feas = lin_fd2 - sel_fd2*invttail(d_lf, 0.025)
		gen ub_d2_feas = lin_fd2 + sel_fd2*invttail(d_lf, 0.025)
		gen cover_feas_lin = lb_d2_feas < 2 & 2 < ub_d2_feas

		gen lb_d2_ap = lin_apd2 - sel_apd2*invttail(d_la, 0.025)
		gen ub_d2_ap = lin_apd2 + sel_apd2*invttail(d_la, 0.025)
		gen cover_ap_lin = lb_d2_ap < 2 & 2 < ub_d2_ap

*Effect of feas and ap on T1

		*Full Probit coefficients
		gen lb_probit_feas = probit_feas - sep_feas*invnormal(0.975)
		gen ub_probit_feas = probit_feas + sep_feas*invnormal(0.975)
		gen cover_probit_feas = lb_probit_feas < 1 & 1 < ub_probit_feas

		gen lb_probit_ap = probit_ap - sep_ap*invnormal(0.975)
		gen ub_probit_ap = probit_ap + sep_ap*invnormal(0.975)
		gen cover_probit_ap = lb_probit_ap < 1 & 1 < ub_probit_ap


* Effect of T1 on Y2

		*Reg short
		gen lb_t1 = b_t1 - se_t1*invttail(df_r, 0.025)
		gen ub_t1 = b_t1 + se_t1*invttail(df_r, 0.025)
```

```
	gen cover_t1 = lb_t1 < atate & atate < ub_t1

	*Reg long
	gen lb_t1e = b_t1e - se_t1e*invttail(df_re, 0.025)
	gen ub_t1e = b_t1e + se_t1e*invttail(df_re, 0.025)
	gen cover_t1e = lb_t1e < atate & atate < ub_t1e

log close
exit, clear
```

References for the Supplementary Material

1. Goldberger AS. Structural equation methods in the social sciences. Econometrica. 1972;40: 979–1001. doi:10.2307/1913851

2. Train KE. Discrete choice methods with simulation. 2nd ed. New York, NY: Cambridge University Press; 2009.

3. Hensher DA, Rose JM, Greene WH. Applied choice analysis. 2nd ed. Cambridge, UK: Cambridge University Press; 2015.

4. Pearl J. Causality: models, reasoning, and inference. 2nd ed. New York, NY: Cambridge University Press; 2009.

5. Frisch RAK. Statistical versus theoretical relations in economic macrodynamics. Paper given at League of Nations. In: Henry DF, Morgan MS, editors. The foundations of econometric analysis. Cambridge, UK: Cambridge University Press; 1995. pp. 407–419.

6. Heckman JJ, Pinto R. Causal analysis after Haavelmo. Econom Theory. 2015;31: 115–151. doi:10.1017/S026646661400022X

7. White H, Lu X. Causal diagrams for treatment effect estimation with application to efficient covariate selection. Rev Econ Stat. 2011;93: 1453–1459. doi:10.1162/REST_a_00153

8. Imbens GW, Rubin, DB. Causal inference for statistics, social, and biomedical sciences: an introduction. New York, NY: Cambridge University Press; 2015.

9. Heckman JJ, Humphries JE, Veramendi G. Dynamic treatment effects. J Econom. 2016;191: 276–292. doi:10.1016/j.jeconom.2015.12.001

10. Cook TD. Twenty-six assumptions that have to be met if single random assignment experiments are to warrant “gold standard” status: a commentary on Deaton and Cartwright. Soc Sci Med. 2018;210: 37–40. doi:10.1016/j.socscimed.2018.04.031

11. Curran GM, Bauer M, Mittman B, Pyne JM, Stetler C. Effectiveness-implementation hybrid designs: combining elements of clinical effectiveness and implementation research to enhance public health impact. Med Care. 2012;50: 217–226. doi:10.1097/MLR.0b013e3182408812

12. Low H, Meghir C. The use of structural models in econometrics. J Econ Perspect. 2017;31: 33–58. doi:10.1257/jep.31.2.33

13. Pearl J, Glymour M, Jewell NP. Causal inference in statistics: a primer. Chichester, UK: John Wiley & Sons Ltd; 2016.

14. Imbens GW, Wooldridge JM. Recent developments in the econometrics of program evaluation. J Econ Lit. 2009;47: 5–86. doi:10.1257/jel.47.1.5

15. Cameron AC, Miller DL. A practitioner’s guide to cluster-robust inference. J Hum Resour. 2015;50: 317–372. doi:10.3368/jhr.50.2.317

16. Wooldridge JM. Econometric analysis of cross section and panel data. 2nd ed. Cambridge, MA: MIT Press; 2010.

17. Cameron AC, Trivedi PK. Microeconometrics: methods and applications. New York, NY: Cambridge University Press; 2005.

18. James A, Soler A, Weatherall R. Cognitive behavioural therapy for anxiety disorders in children and adolescents. Cochrane Database Syst Rev. 2005; doi:10.1002/14651858.CD004690.pub2

19. Hofmann SG, Asnaani A, Vonk IJJ, Sawyer AT, Fang A. The Efficacy of cognitive behavioral therapy: a review of meta-analyses. Cogn Ther Res. 2012;36: 427–440. doi:10.1007/s10608-012-9476-1

20. James AC, James G, Cowdrey FA, Soler A, Choke A. Cognitive behavioural therapy for anxiety disorders in children and adolescents. Cochrane Database Syst Rev. 2015; doi:10.1002/14651858.CD004690.pub4

21. Aarons GA, Ehrhart MG, Farahnak LR. The implementation leadership scale (ILS): development of a brief measure of unit level implementation leadership. Implement Sci. 2014;9: 45. doi:10.1186/1748-5908-9-45

22. Burton A, Altman DG, Royston P, Holder RL. The design of simulation studies in medical statistics. Stat Med. 2006;25: 4279–4292. doi:10.1002/sim.2673

23. Bryan ML, Jenkins SP. Multilevel modelling of country effects: a cautionary tale. Eur Sociol Rev. 2016;32: 3–22. doi:10.1093/esr/jcv059

24. Lewis CC, Scott K, Marti CN, Marriott BR, Kroenke K, Putz JW, et al. Implementing measurement-based care (iMBC) for depression in community mental health: a dynamic cluster randomized trial study protocol. Implement Sci. 2015;10: 127. doi:10.1186/s13012-015-0313-2
